# Supplementary material for: Evaluation of dihydropyranocoumarins as potent inhibitors against triple-negative breast cancer: An integrated of in silico, quantum & molecular modeling approaches
Source: PLoS One. 2025 Dec 3;20(12):e0334939. doi: 10.1371/journal.pone.0334939 (PMC12674555; doi:10.1371/journal.pone.0334939)
Supplement: S1 Table — (DOCX) [file pone.0334939.s001.docx]

**Table S1*.* Data for redocking procedure by control**

|  | Binding Affinity  (kcal/mol) | RMSD | No of H Bond | No of Hydrophobic Bond |
| --- | --- | --- | --- | --- |
| Control 1 for Human CK2 alpha kinase (PUB ID 7L1X) | -8.3 | 0 | 6 | 1 |
| Control 2 for TNBC receptor  (PUB ID 5HA9) | -9.0 | 0 | 0 | 4 |
