# Supplementary material for: Evaluation of dihydropyranocoumarins as potent inhibitors against triple-negative breast cancer: An integrated of in silico, quantum & molecular modeling approaches
Source: PLoS One. 2025 Dec 3;20(12):e0334939. doi: 10.1371/journal.pone.0334939 (PMC12674555; doi:10.1371/journal.pone.0334939)
Supplement: S2 Table — (DOCX) [file pone.0334939.s002.docx]

**Table S2: Protein ligand interaction data**

| Ligand | CK2 Alpha Kinase (7L1X) | | | | TNBC Receptor (5HA9) | | | |
| --- | --- | --- | --- | --- | --- | --- | --- | --- |
|  | **Hydrogen Bond** | **Distance (Å)** | **Hydrophobic Bond** | **Distance (Å)** | **Hydrogen Bond** | **Distance (Å)** | **Hydrophobic Bond** | **Distance (Å)** |
| Isosamidin | Absent | Absent | VAL-53  VAL-66  MET- 163  ILE- 174  VAL- 53  LYS- 68  ILE- 174 | 5.43  4.06  5.47  4.16  4.60  5.04  4.09 | ASP- 129  ILE- 147 | 2.26  3.52 | VAL- 184  VAL- 184 | 3.74  3.7 |
| Pteryxin | Absent | Absent | VAL-53  VAL-66  ILE- 174  VAL- 53  LYS- 68  ILE- 174 | 5.01  4.11  4.30  4.55  5.13  4.06 | ASP- 129 | 2.49 | VAL- 184  VAL- 184 | 3.55  3.81 |
| Suksdorfin | Absent | Absent | MET- 163  VAL- 53  VAL- 66  MET- 163  ILE- 174  VAL- 53  LYS- 68  ILE- 174 | 4.91  5.33  4.09  5.38  4.17  4.59  5.11  4.05 | ASP- 129  ILE- 147 | 2.38  3.66 | VAL- 184  VAL- 184 | 3.60  3.69 |
| Visnadine | Absent | Absent | VAL- 53  VAL- 66  MET- 163  ILE- 174  VAL- 53  LYS- 68  ILE- 174 | 5.33  4.06  5.40  4.20  4.55  5.08  4.10 | ASP- 129  ILE- 147 | 2.39  3.54 | VAL- 184  VAL- 184 | 3.57  3.81 |
