# Supplementary material for: Evaluation of dihydropyranocoumarins as potent inhibitors against triple-negative breast cancer: An integrated of in silico, quantum & molecular modeling approaches
Source: PLoS One. 2025 Dec 3;20(12):e0334939. doi: 10.1371/journal.pone.0334939 (PMC12674555; doi:10.1371/journal.pone.0334939)
Supplement: S3 Table — (DOCX) [file pone.0334939.s003.docx]

**Table S3: Growth and serum parameters of in vivo studies**

| Parameters | Experimental diet groups | | | |
| --- | --- | --- | --- | --- |
|  | Experiment 1 | | Experiment 2 | |
|  | Control | PJT-PTX (+) | Control | PJT-PTX (−) |
| Food intake (g/day) | 2.64 ± 0.06 | 2.58 ± 0.09 | 2.58 ± 0.06 | 2.58 ± 0.05 |
| Energy intake (kcal/day) | 12.2 ± 0.3 | 11.9 ± 0.4 | 11.9 ± 0.3 | 11.9 ± 0.2 |
| Initial body weight (g) | 18.0 ± 0.3 | 17.7 ± 0.3 | 15.3 ± 0.3 | 15.3 ± 0.3 |
| Final body weight (g) | 25.2 ± 0.4 | 22.3 ± 0.5** | 24.4 ± 0.6 | 21.6 ± 0.4** |
| *Serum parameter* |  |  |  |  |
| TG (mg/dL) | 50.1 ± 9.8 | 40.7 ± 5.2 | 97.9 ± 14.6 | 72.8 ± 10.3 |
| TC (mg/dL) | 104 ± 4 | 109 ± 6 | 119 ± 11 | 133 ± 6 |
| FFA (mg/dL) | 1.50 ± 0.12 | 1.21 ± 0.07 | 1.81 ± 0.16 | 1.23 ± 0.08* |
| Leptin (ng/mL) | 2.53 ± 0.43 | 1.42 ± 0.17 | 3.92 ± 1.28 | 1.44 ± 0.41 |
| ALT (IU/L) | 25.4 ± 3.1 | 26.5 ± 1.5 | 31.7 ± 3.9 | 22.3 ± 2.5 |
| AST (IU/L) | 10.5 ± 0.3 | 10.6 ± 0.2 | 5.58 ± 0.67 | 4.75 ± 6.7 |

* TG: triglyceride; TC: total cholesterol; FFA: free fatty acid; ALT: alanine aminotransferase; AST: aspartate aminotransferase.
