# Supplementary material for: Evaluation of dihydropyranocoumarins as potent inhibitors against triple-negative breast cancer: An integrated of in silico, quantum & molecular modeling approaches
Source: PLoS One. 2025 Dec 3;20(12):e0334939. doi: 10.1371/journal.pone.0334939 (PMC12674555; doi:10.1371/journal.pone.0334939)
Supplement: S1 Fig — (DOCX) [file pone.0334939.s004.docx]

| 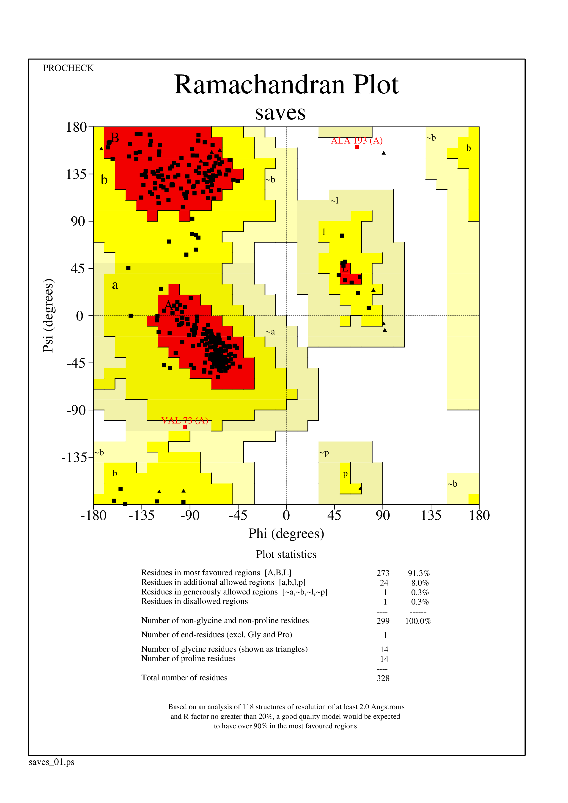 | 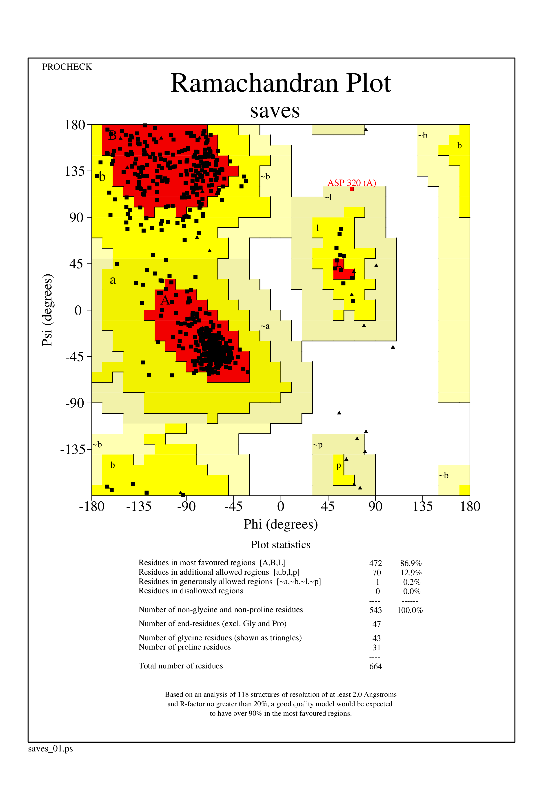 |
| --- | --- |
| A | B |

**S1 Fig. Ramachandran plots of (A) PARP1 (PDB ID: 5HA9) and (B) CK2α (PDB ID: 7L1X) generated using PROCHECK.**

The stereochemical quality of the selected protein structures (PDB IDs: 5HA9 and 7L1X) was evaluated using PROCHECK, which generates Ramachandran plots. For 5HA9, 86.9% of residues were located in the most favoured regions and 12.9% in the additionally allowed regions, with no residues in generously allowed or disallowed regions (Figure X). For 7L1X, 91.3% of residues were located in the most favoured regions and 8.0% in the additionally allowed regions, with no residues in generously allowed or disallowed regions (Figure X). These results confirm the high stereochemical quality of both protein structures, supporting their suitability for docking studies.
