# Supplementary material for: Evaluation of dihydropyranocoumarins as potent inhibitors against triple-negative breast cancer: An integrated of in silico, quantum & molecular modeling approaches
Source: PLoS One. 2025 Dec 3;20(12):e0334939. doi: 10.1371/journal.pone.0334939 (PMC12674555; doi:10.1371/journal.pone.0334939)
Supplement: S2 Fig — (DOCX) [file pone.0334939.s005.docx]

| 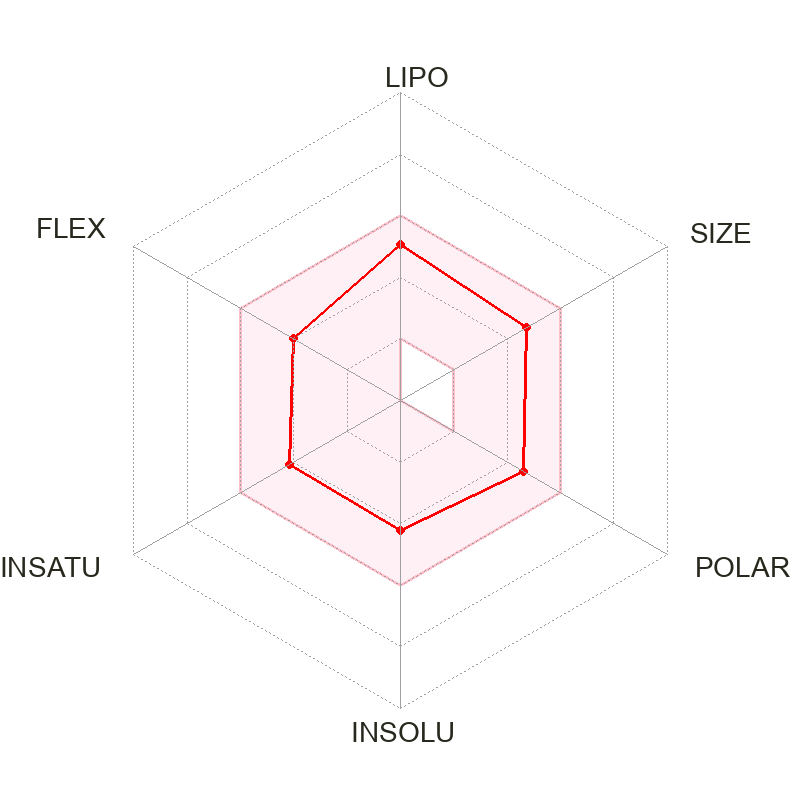  L01 | 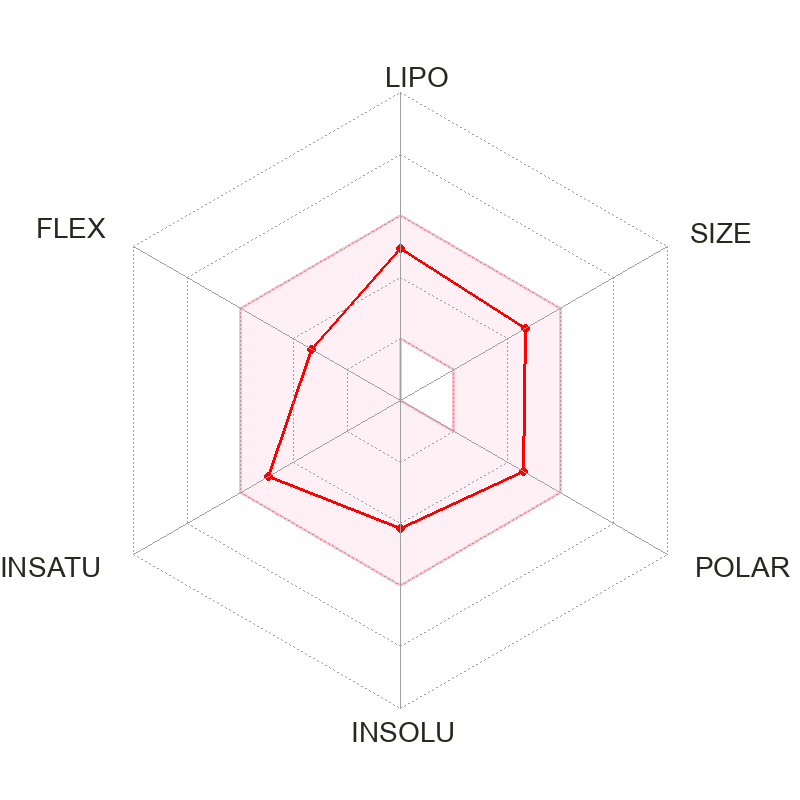  L02 |
| --- | --- |
| 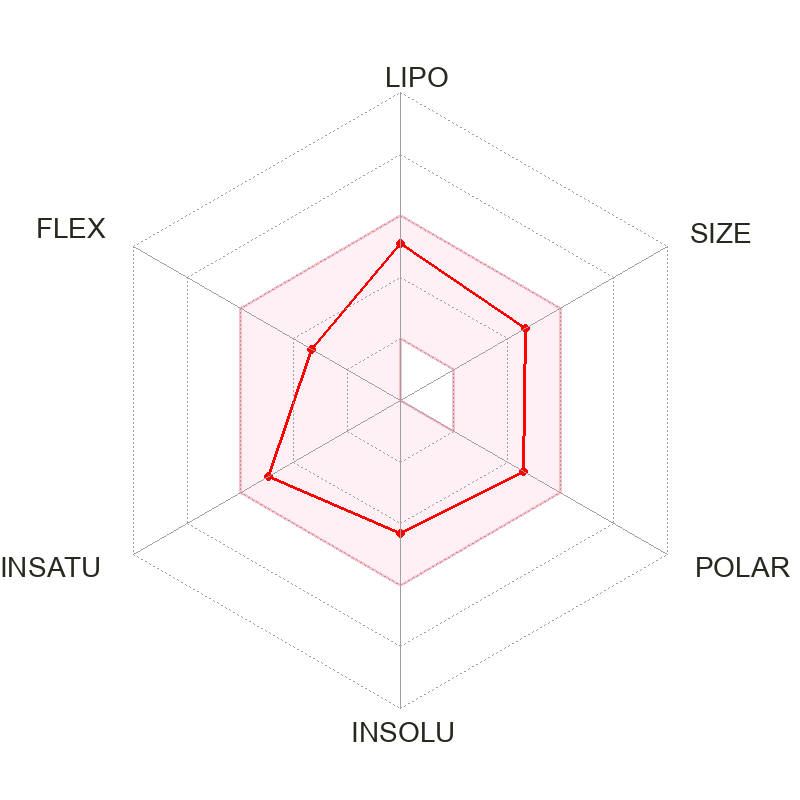  L03 | 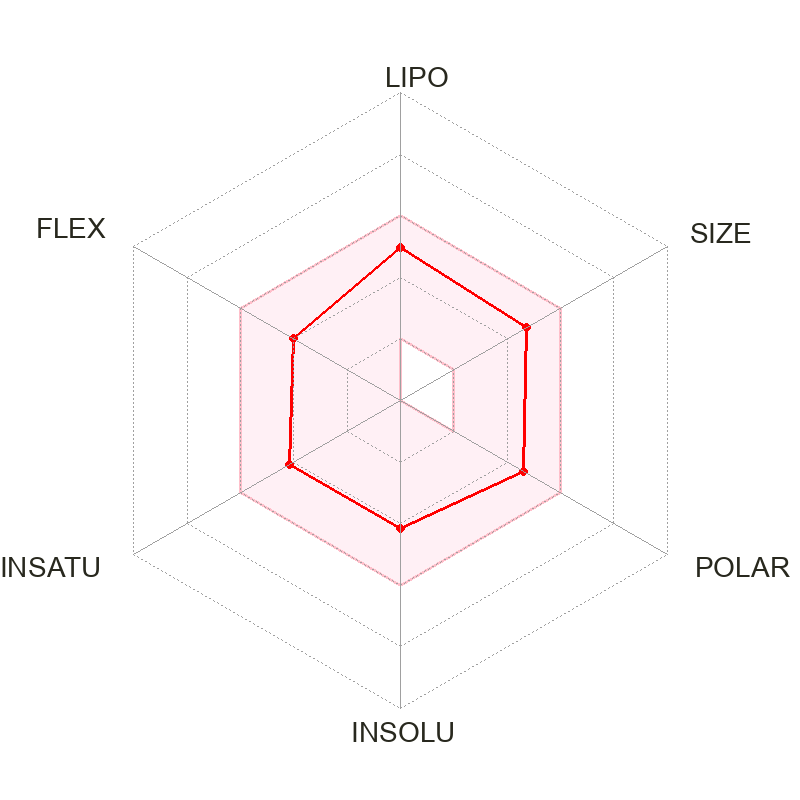  L04 |
| 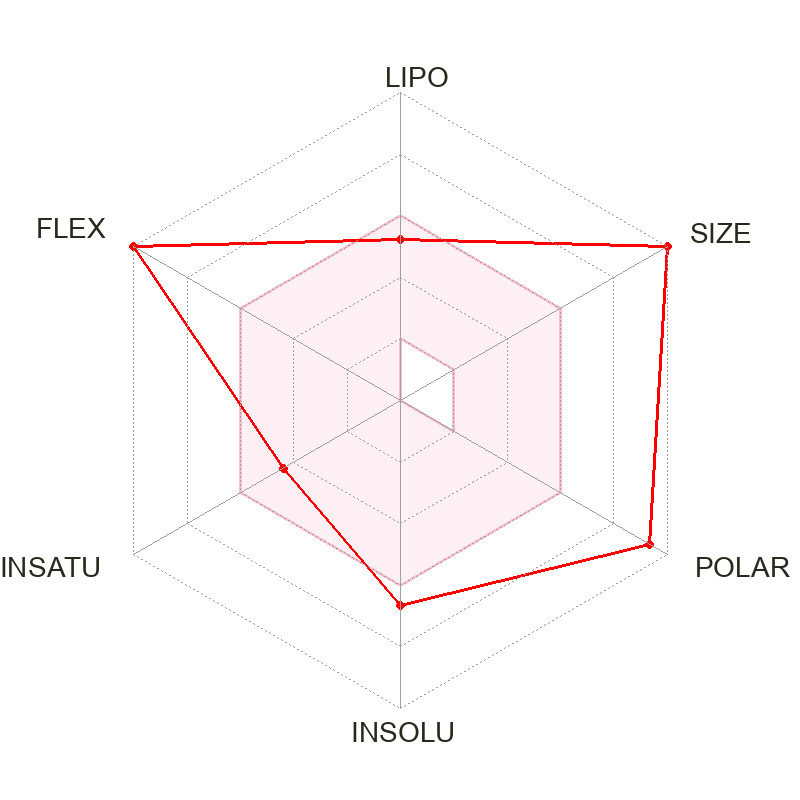  Paclitaxel | 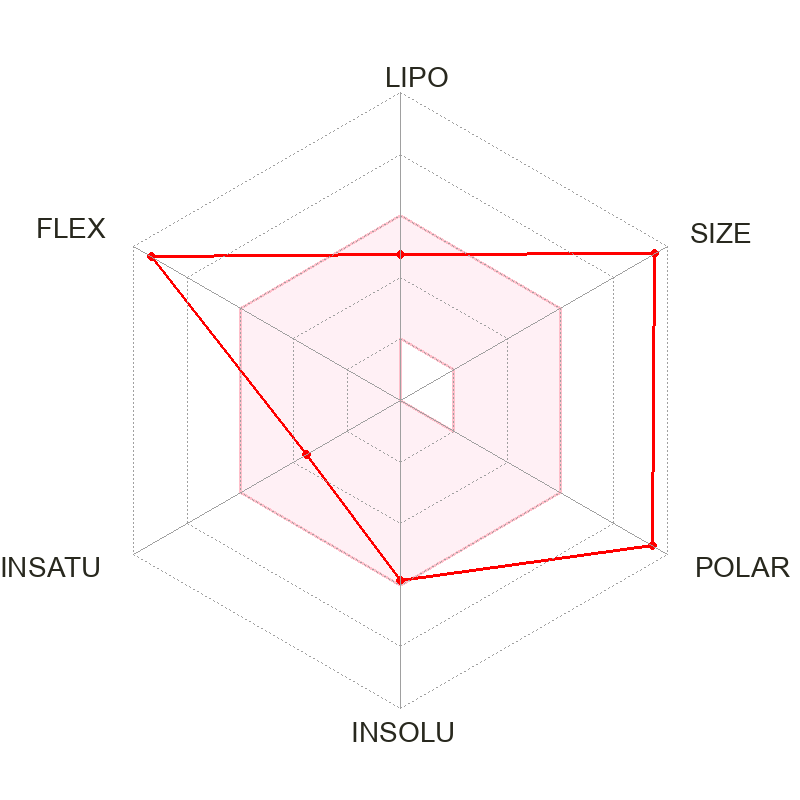  Docetaxel |
| 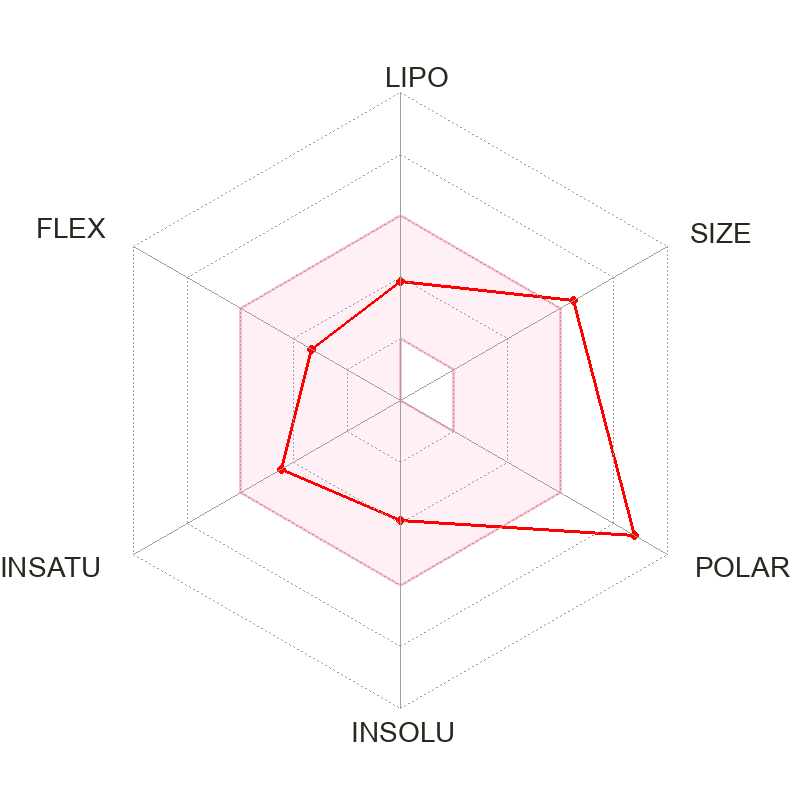  Doxorubicin | 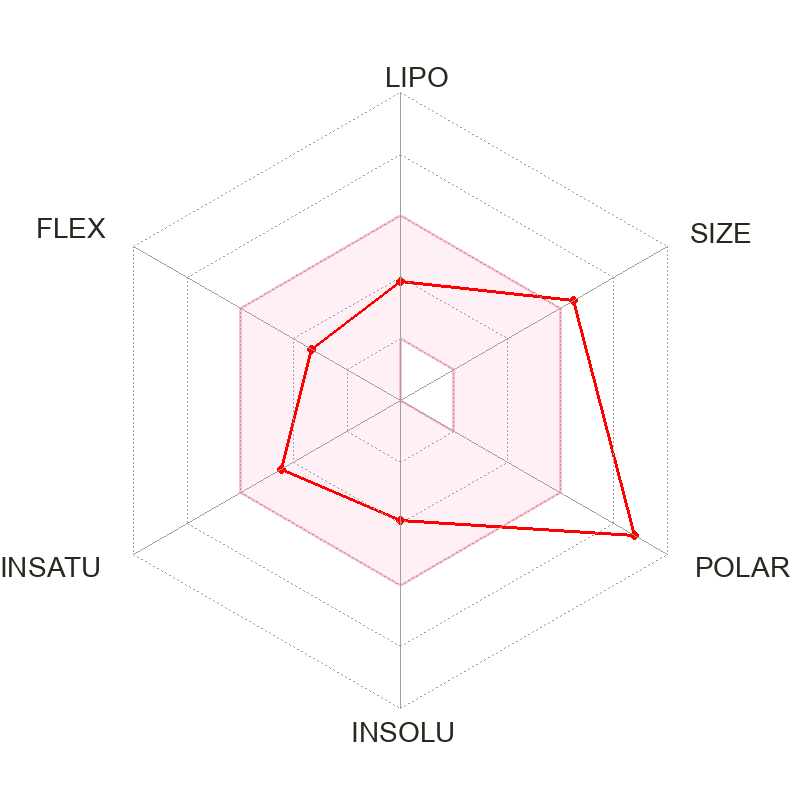  Epirubicin |

**S2 Fig. Bioavailability radar plots for PJT-derived ligands (L01–L04) and known TNBC drugs (Paclitaxel, Docetaxel, Doxorubicin, Epirubicin).**
