# Supplementary material for: Evaluation of dihydropyranocoumarins as potent inhibitors against triple-negative breast cancer: An integrated of in silico, quantum & molecular modeling approaches
Source: PLoS One. 2025 Dec 3;20(12):e0334939. doi: 10.1371/journal.pone.0334939 (PMC12674555; doi:10.1371/journal.pone.0334939)
Supplement: S3 Fig — (DOCX) [file pone.0334939.s006.docx]

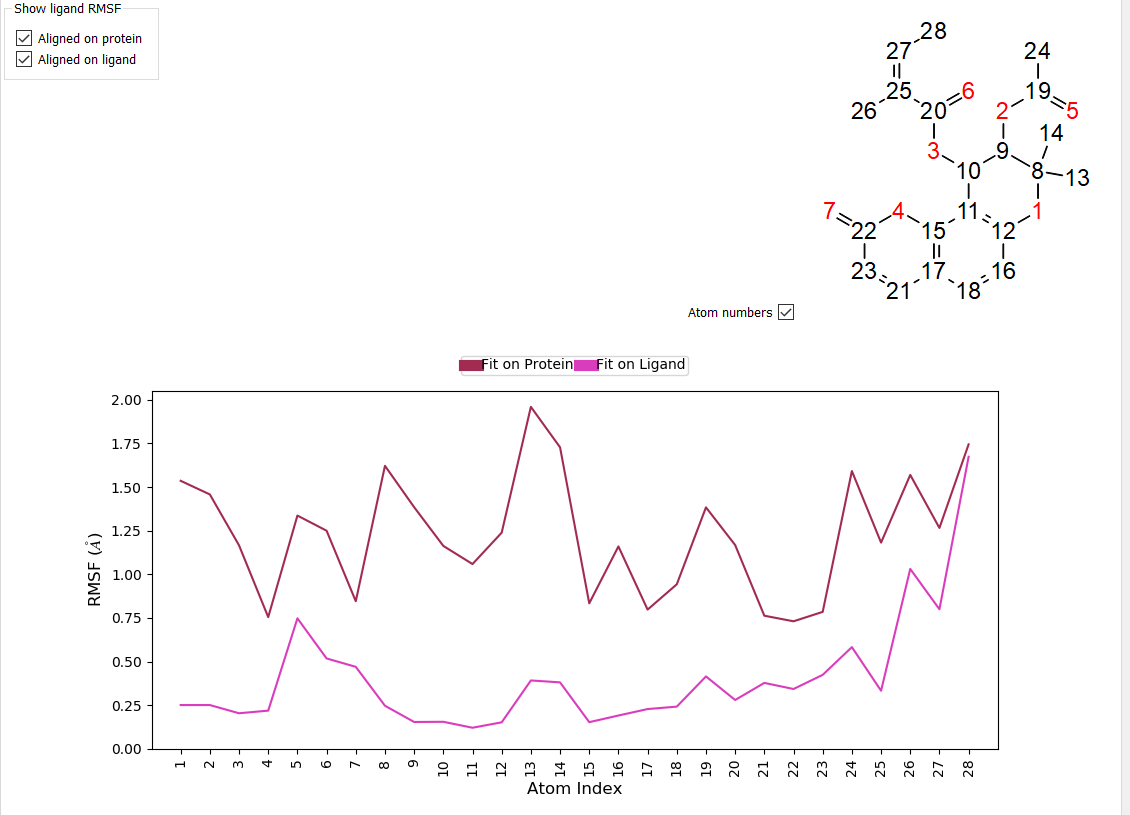


**S3 Fig. The graph titled "L-RMSF" (Ligand Root Mean Square Fluctuation) illustrates the flexibility of a ligand, with the x-axis representing atom indices (e.g., Aton, Bton, Cton, Dton) and the y-axis showing RMSF values in Ångströms (Å).**

The graph likely includes two lines: one where the ligand is aligned on the protein (possibly in blue) and another where the ligand is aligned on itself (possibly in red). Higher RMSF values indicate greater flexibility, while lower values suggest rigidity. For example, atom indices 10-20 might show RMSF values around 0.8 Å when aligned on the protein and 1.2 Å when aligned on the ligand, indicating slight differences in flexibility based on alignment. Peaks at specific atom indices, such as atom 30 (1.5 Å) or atom 50 (2.0 Å), highlight regions of high mobility, possibly due to flexible bonds or side chains. This graph helps analyze the ligand's dynamic behavior and how its flexibility varies depending on the alignment method, providing insights into its interaction with the protein.
