# Supplementary material for: Evaluation of dihydropyranocoumarins as potent inhibitors against triple-negative breast cancer: An integrated of in silico, quantum & molecular modeling approaches
Source: PLoS One. 2025 Dec 3;20(12):e0334939. doi: 10.1371/journal.pone.0334939 (PMC12674555; doi:10.1371/journal.pone.0334939)
Supplement: S4 Fig — (DOCX) [file pone.0334939.s007.docx]

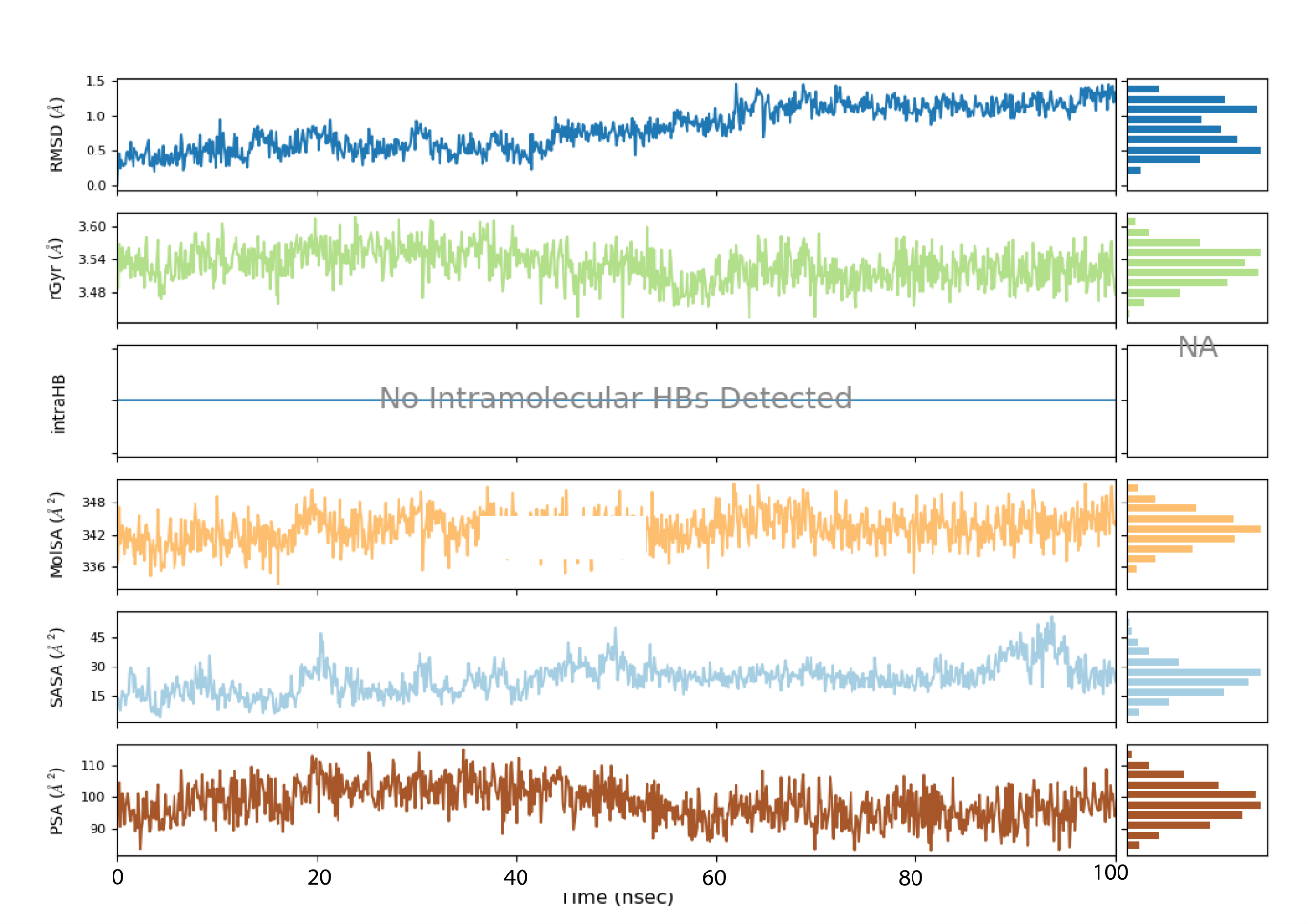


**S4 Fig.** **Radius of gyration (Rg) of WT, mutations, Solvent-accessible surface area (SASA).**

The graph titled "L-Properties" visualizes various properties of a ligand over time, with the x-axis representing time in nanoseconds (nsec) and the y-axis showing different property values. The properties include **RSI (d)** (possibly in blue), **RV (h)** (possibly in red), **Toyr (h)** (possibly in green), **SASA (i²)** (solvent-accessible surface area, possibly in purple), and **PSA (i²)** (polar surface area, possibly in orange). The graph indicates that no intramolecular hydrogen bonds (HBs) were detected. For example, RSI values might fluctuate between 0.5 and 1.5, RV values between 10 and 20, Toyr values between 5 and 15, SASA values between 100 and 200 i², and PSA values between 50 and 100 i² over the 100 nsec simulation. Specific time points, such as 20 nsec (RSI: 1.0, RV: 15, Toyr: 10, SASA: 150, PSA: 75) and 60 nsec (RSI: 1.2, RV: 18, Toyr: 12, SASA: 180, PSA: 80), highlight how these properties evolve. This graph provides insights into the ligand's dynamic behavior, including its flexibility, solvent accessibility, and polarity over time.
