# Supplementary material for: Evaluation of dihydropyranocoumarins as potent inhibitors against triple-negative breast cancer: An integrated of in silico, quantum & molecular modeling approaches
Source: PLoS One. 2025 Dec 3;20(12):e0334939. doi: 10.1371/journal.pone.0334939 (PMC12674555; doi:10.1371/journal.pone.0334939)
Supplement: S5 Fig — (DOCX) [file pone.0334939.s008.docx]

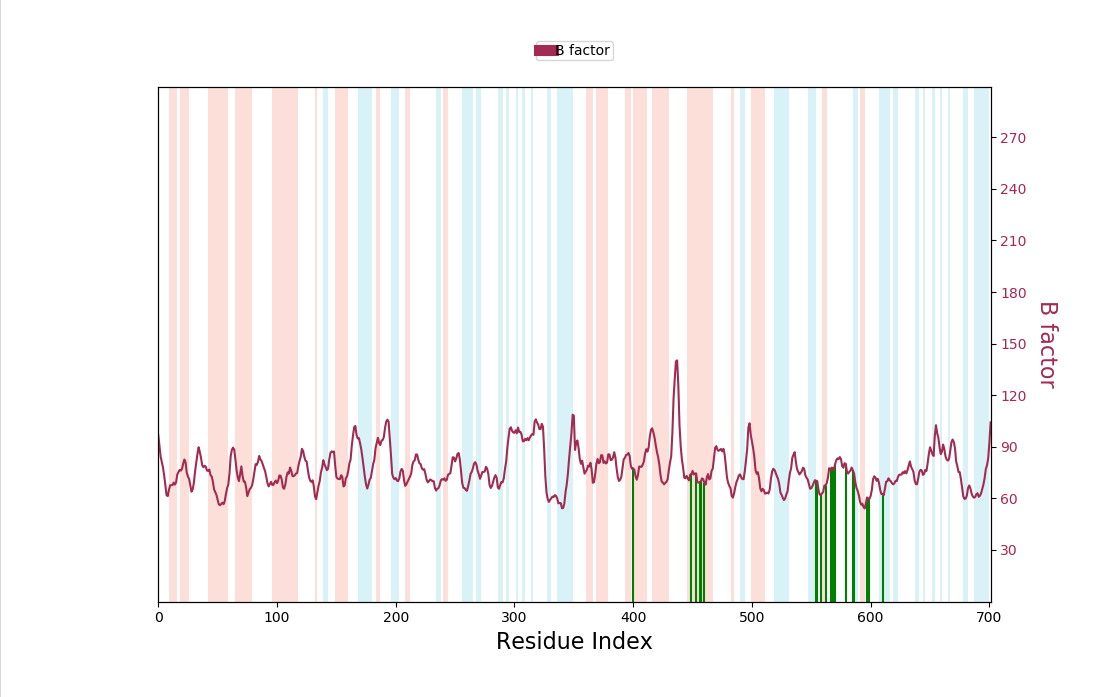


**S5 Fig. The graph titled "P-RMSF B factor" visualizes the Root Mean Square Fluctuation (RMSF) and B-factor (temperature factor) of a protein, with the x-axis representing the residue index (ranging from 0 to 700) and the y-axis showing the B-factor values.**

The B-factor indicates the flexibility or mobility of each residue, with higher values (e.g., 30, 60, 90) suggesting greater flexibility and lower values indicating rigidity. The graph likely uses a color gradient or line plot to represent the B-factor, with peaks at specific residues highlighting regions of high mobility. For example, residues 100-150 might show B-factors around 60, while residues 200-250 could peak at 90, indicating increased flexibility in those regions. Specific residues, such as residue 300 (B-factor: 120) or residue 400 (B-factor: 150), may stand out as highly dynamic, possibly due to loops or unstructured regions. This graph helps identify flexible and stable regions of the protein, providing insights into its structural dynamics and potential functional sites.
