# Supplementary material for: Evaluation of dihydropyranocoumarins as potent inhibitors against triple-negative breast cancer: An integrated of in silico, quantum & molecular modeling approaches
Source: PLoS One. 2025 Dec 3;20(12):e0334939. doi: 10.1371/journal.pone.0334939 (PMC12674555; doi:10.1371/journal.pone.0334939)
Supplement: S6 Fig — (DOCX) [file pone.0334939.s009.docx]

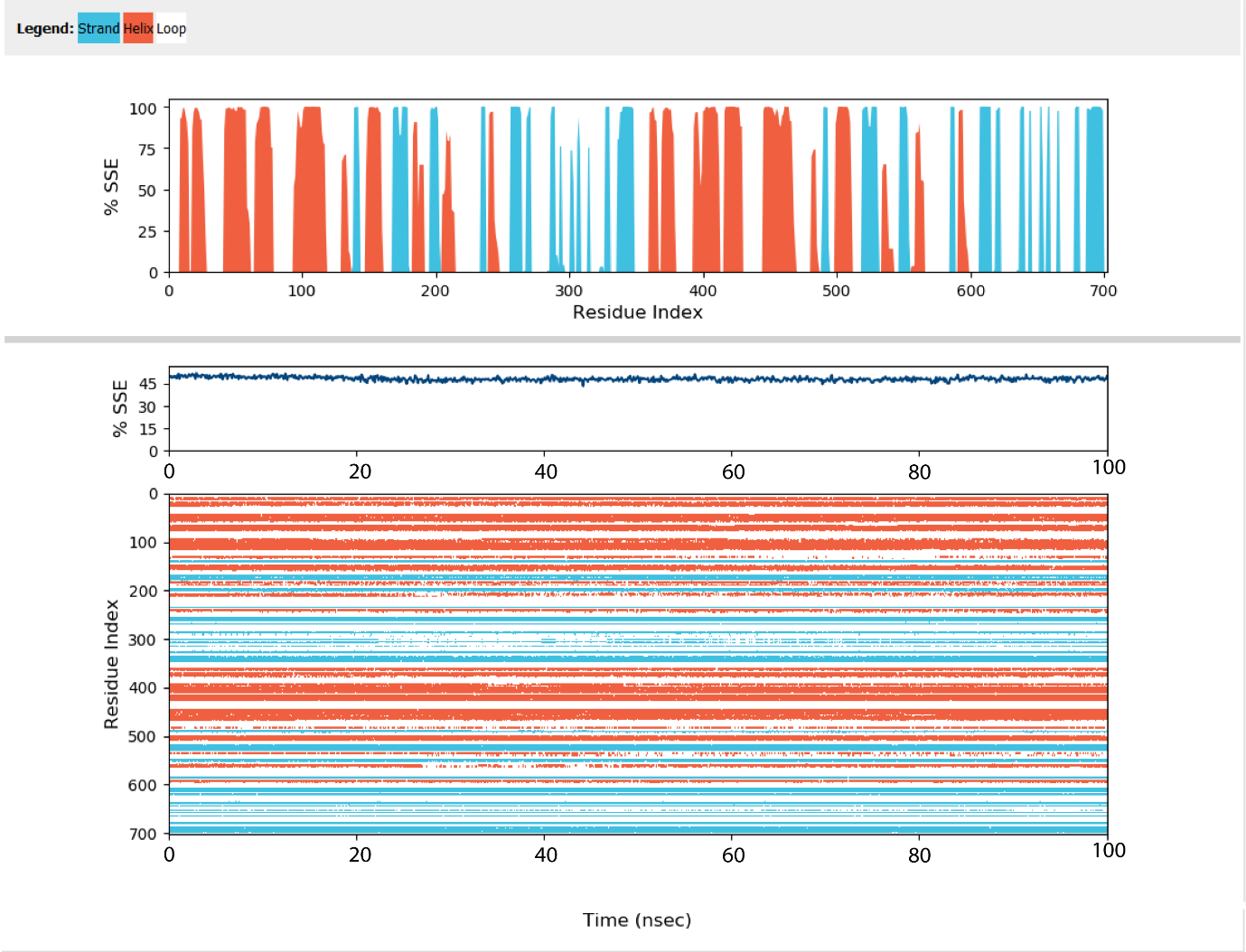


**S6 Fig. Protein secondary structure elements (SSE) of protein- ligand complexes.**

The graph titled "PSS" (Protein Secondary Structure) illustrates the secondary structure elements of a protein over time, with the x-axis representing the residue index (ranging from 200 to 700) and the y-axis showing the time in nanoseconds (nsec). The graph likely uses different colors to represent secondary structure elements: **Helix** (possibly in red), **Strand** (possibly in blue), and **Loop** (possibly in green). The y-axis values (e.g., LH: 100, 75, 50, 25, 0 and SG: 45, 30, 15, 0) indicate the prevalence or stability of these elements. For example, residues 300-400 might show high helix content (LH: 75) and low strand content (SG: 15), while residues 500-600 could have more loops (green regions). Specific time points, such as 20 nsec (residue 250: Helix 50, Strand 30) and 60 nsec (residue 450: Helix 25, Strand 45), highlight how the secondary structure evolves. This graph helps analyze the stability and transitions of secondary structures, providing insights into the protein's conformational dynamics over time.
