# Supplementary figures and images for: Evaluation of dihydropyranocoumarins as potent inhibitors against triple-negative breast cancer: An integrated of in silico, quantum & molecular modeling approaches
Source: PLoS One. 2025 Dec 3;20(12):e0334939. doi: 10.1371/journal.pone.0334939 (PMC12674555; doi:10.1371/journal.pone.0334939)

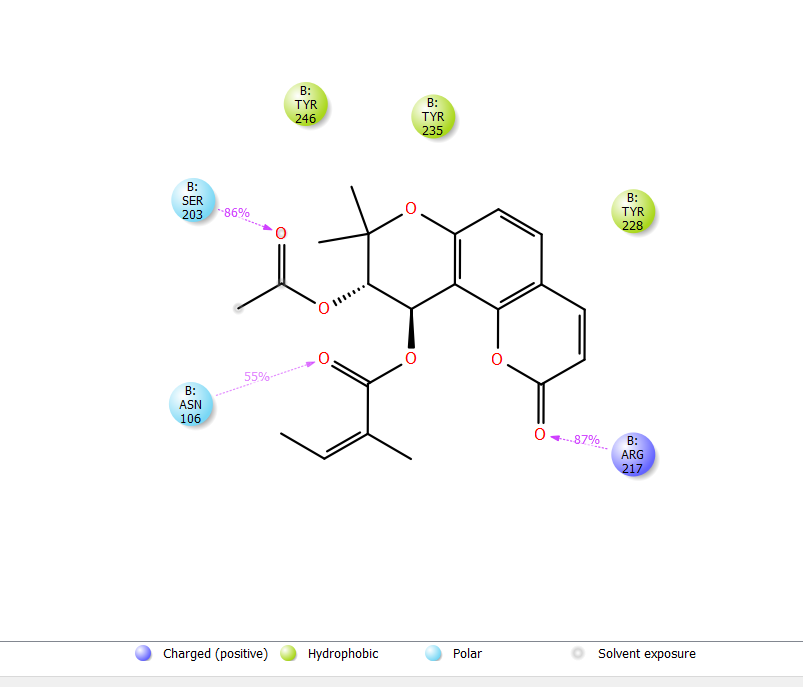


**S7 Fig. Molecular interaction between the ligand and target**

Supplement: S7 Fig — (DOCX) [file pone.0334939.s010.docx]

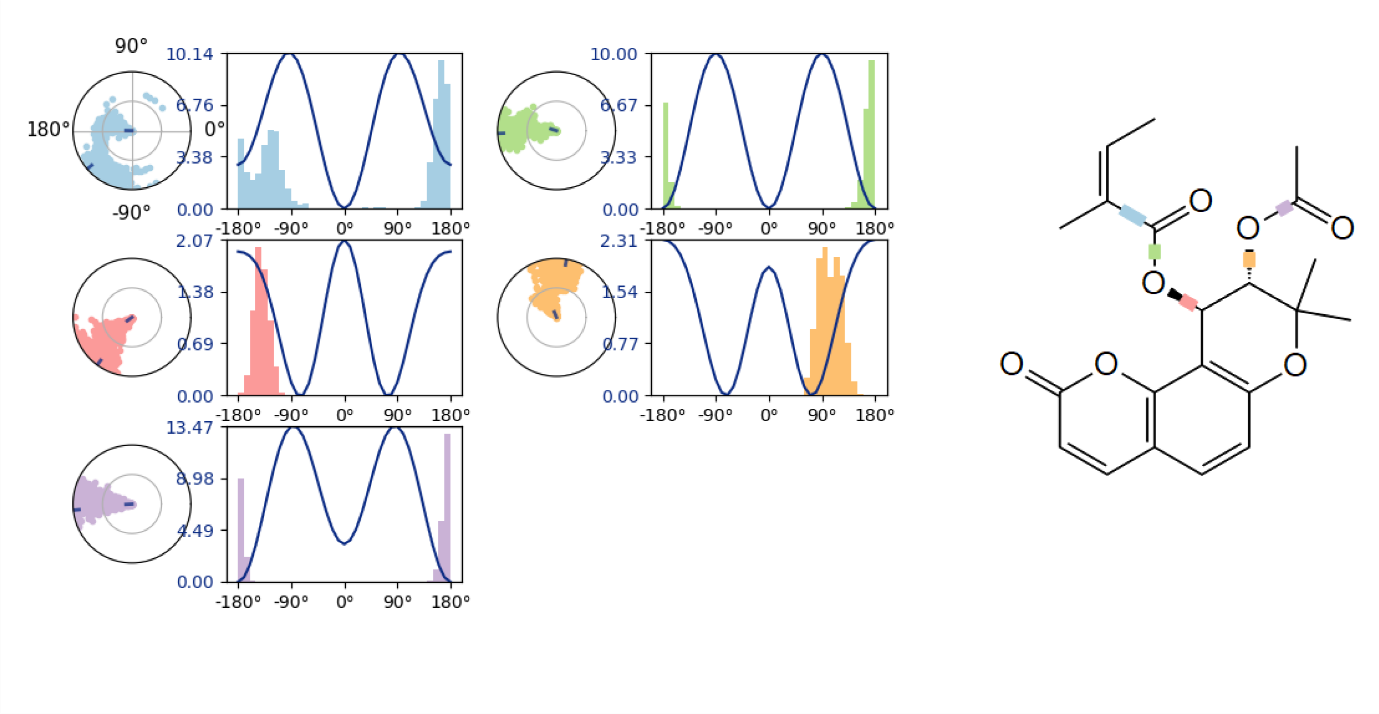


**S8 Fig. Torsional flexibility In Molecular Docking Studies protein- ligand complexes.**

Supplement: S8 Fig — (DOCX) [file pone.0334939.s011.docx]
