# Supplementary material for: Evaluation of dihydropyranocoumarins as potent inhibitors against triple-negative breast cancer: An integrated of in silico, quantum & molecular modeling approaches
Source: PLoS One. 2025 Dec 3;20(12):e0334939. doi: 10.1371/journal.pone.0334939 (PMC12674555; doi:10.1371/journal.pone.0334939)
Supplement: Attachment — Submitted filename: Ikechukwu kanu Review new 1.pdf [file pone.0334939.s012.pdf]

Dear Esteemed Editor,

I have examined the publication entitled " Evaluation of Dihydropyranocoumarins as Potent Inhibitors Against Triple-Negative Breast Cancer: An Integrated DFT, in silico approaches, Docking and MD Study. This publication examines the combined anti-cancer efficacy of ONC201 and MAPK pathway inhibitors (trametinib, ulixertinib, and AMG-510) in triple-negative breast cancer (TNBC). The research indicates that ONC201 triggers apoptosis in TNBC cells and, when used in conjunction with MAPK inhibitors, markedly increases cell death and suppresses tumor growth both in vitro and in vivo. The authors propose that dual targeting of ERK signaling, and ClpP/ATF4-mediated apoptosis is a viable treatment approach for TNBC. However, I have identified several areas needing modification before the manuscript may be recommended for publication.

Strengths:

Scope and Relevance:

This research addresses a significant unmet requirement in TNBC, a subtype devoid of effective targeted treatments.
It also evaluates ONC201, a small drug presently undergoing clinical review, in a strategic combination therapy with MAPK pathway inhibitors.
The notion of synthetic lethality via mitochondrial ClpP activation and ERK pathway inhibition is innovative and clinically significant.

Methodology:
The authors utilized appropriate assays: Cell viability, Western blot, apoptosis, qRT-PCR, colony formation, and animal models. Several inhibitors (trametinib, ulixertinib, AMG-510) were utilized to analyze the components of the MAPK pathway involved in synergy.

Presentation:

The manuscript presents a coherent and compelling rationale for targeting TNBC using a dual strategy involving ONC201 and MAPK inhibitors. Also, each section (Introduction, Methods, Results, Discussion) flows logically and supports the central hypothesis.

Suggestions:

Numerous figure legends and result descriptions require more detail, such as the specification of doses and time points utilized in investigations.

Certain acronyms (e.g., ISR, ClpP) are employed without prior elucidation.

Several statements in the Discussion section are excessively lengthy or conjectural and might benefit explanation.

In the Results, explicitly differentiate between in vitro and in vivo observations.

Limitations:

The Synergism between ONC201 and MAPK inhibitors is described but lacks formal synergy quantification.

Only a single xenograft model (MDA-MB-231) is used and no histopathological or IHC analysis of tumors to confirm pathway inhibition or apoptosis markers in vivo.

Some bar graphs lack error bars or significance indicators.

.
