# Supplementary material for: Evaluation of dihydropyranocoumarins as potent inhibitors against triple-negative breast cancer: An integrated of in silico, quantum & molecular modeling approaches
Source: PLoS One. 2025 Dec 3;20(12):e0334939. doi: 10.1371/journal.pone.0334939 (PMC12674555; doi:10.1371/journal.pone.0334939)
Supplement: Attachment — Submitted filename: Response to Reviewer R1.docx [file pone.0334939.s013.docx]

PONE-D-25-33604R1

Evaluation of Dihydropyranocoumarins as Potent Inhibitors Against Triple-Negative Breast Cancer: An Integrated of In silico, Quantum & Molecular Modeling Approaches

Dear Dr. Kumer,

We've checked your submission and before we can proceed, we need you to address the following issues:

1. Please provide a complete Data Availability Statement in the submission form, ensuring you include all necessary access information or a reason for why you are unable to make your data freely accessible. If your research concerns only data provided within your submission, please write "All data are in the manuscript and/or supporting information files" as your Data Availability Statement.

***Answer: It has revised and added.***

2. Please ensure that you refer to Figure 5, 6, and 7 in your text as, if accepted, production will need this reference to link the reader to the figures.

***Answer: It has revised and added.***

3. Please include a copy of Table 1-8 which you refer to in your manuscript file.

***Answer: It has revised and added.***

4. Please upload a Response to Reviewers letter which should include a point-by-point response to each of the points made by the Editor and / or Reviewers. (This should be uploaded as a 'Response to Reviewers' file type.) Please follow this link for more information: http://blogs.PLOS.org/everyone/2011/05/10/how-to-submit-your-revised-manuscript/

***Answer: It has revised and added.***

5. We notice that your revision was submitted on [Sep 11 2025], but the manuscript file in your submission's file inventory was uploaded on [Jul 1 2025]. Please upload the latest version of your revised manuscript as the main article file, with the item type 'Manuscript,' ensuring that it does not contain any tracked changes or highlighting. This will be used in the production process if your manuscript is accepted.

***Answer: It has revised and added.***

**Response to the Editor and Reviewers**

Manuscript ID: **PONE-D-25-33604**

Title: **Evaluation of Dihydropyranocoumarins as Potent Inhibitors Against Triple-Negative Breast Cancer: An Integrated DFT, in silico approaches, Docking and MD Study**

We sincerely thank the Academic Editor and all reviewers for their valuable time and insightful comments. We have revised our manuscript thoroughly and addressed all comments point by point below. All changes have been incorporated into the revised version, with tracked changes indicated. We believe these revisions have significantly improved the quality and clarity of our manuscript.

**Academic Editor Comment**

**Q1.** The submission reflects scientific relevance. However, some fundamental issues limit its quality for publication in the current form. For instance, the authors need to justify the significance of the study, relate it to the literature and identify the gap in the existing knowledge that this aims to satisfy, and ensure an adequate validation of the theoretical analysis. Again, what are the limitations of this study and how can the authors recommend future research on the study. Moreover, some concerns have been raised by the reviewers affecting certain sections of the study. Kindly pay a thorough attention to these and address them critically before resubmission.

***Response***

*We thank the Academic Editor for highlighting the need to more clearly justify the significance of our study, identify the literature gap, articulate the study’s contribution, and provide adequate validation and future research direction.*

*In response, we have thoroughly revised the Introduction section to explicitly outline the unmet clinical challenges of TNBC, the limitations of current therapeutic strategies, and the promising role of natural products particularly dihydropyranocoumarins from Peucedanum japonicum Thunb. in anticancer drug discovery. We emphasized that despite the pharmacological interest in this plant, no prior research has systematically evaluated its dihydropyranocoumarin derivatives against TNBC targets using a comprehensive in silico strategy. This allowed us to clearly position our work within the existing body of literature and define the knowledge gap our study aims to address.*

*Furthermore, we have explicitly stated our research contribution, which involves the first integrated computational pipeline using DFT calculations, molecular docking, ADMET screening, and 100-ns MD simulations to assess the anti-TNBC potential of these compounds against two clinically relevant TNBC targets (PARP1 and CK2α). This approach provides mechanistic insight into their therapeutic relevance and offers a rational basis for further preclinical development.*

*To ensure balanced interpretation, we have added a disclaimer in the conclusion section clarifying that our findings are predictive and require experimental validation, such as in vitro cytotoxicity assays and in vivo studies. Additionally, we briefly outlined the limitations of our in-silico approach and proposed specific directions for* ***future research****, including experimental validation and formulation strategies to address solubility issues.*

*We have carefully considered all the concerns raised by the reviewers and addressed each point thoroughly in the revised manuscript. We critically reviewed the comments to improve the quality and clarity of our work before resubmission. We believe these revisions have significantly strengthened the paper and hope it now meets the standards for publication.*

**Reviewer #1**

**Q2**. The manuscript is well written and the diagrams are well made and well explained, the concepts are explained thoroughly with providing all the background data that is being used which is good for the person who wants to replicate the research or needs background data.
the tables are also well explained in the passage.

***Response***

*Thank you for the positive feedback. We have preserved the clarity and added further improvement in consistency and figure references where applicable.*

**Reviewer #2**

**Comment**

**1. Overview**

**Q3** (1.I). The manuscript investigates potential new treatment strategies for TNBC, which is a clinically challenging subtype of cancer with unmet therapeutic needs. Targeting it with natural product derivatives is a promising strategy.

***Response***

*First of all, TNBC lacks effective molecular-targeted therapies, making it a priority for novel computational screening approaches. This maintains focus on the computational rationale without overstating clinical aspects.*

*Secondly, Natural product derivatives are ideal for in silico TNBC studies due to their structural diversity, allowing efficient virtual screening and docking. Their known safety profiles and documented biological activity facilitate prioritization and translational potential. Additionally, computational analysis enables rational optimization of functional groups, reducing experimental trial-and-error and accelerating early-stage drug discovery.*

*Thus, TNBC remains a subtype with limited targeted therapies. Computational screening of natural product derivatives allows rapid evaluation of molecular interactions, electronic properties, and pharmacokinetic potential, providing a cost-effective and rational approach to identify promising candidates. Their inherent structural diversity and generally favorable safety profiles make natural products particularly suitable for early-stage computational drug discovery and subsequent translational studies.*

**Q4**  **(1.II).** The manuscript seeks to fill a critical knowledge gap on a clinically important topic, but will benefit from a revision across the areas described below.

***Response***

*We sincerely thank the reviewer for recognizing the clinical importance of triple-negative breast cancer (TNBC) and the promise of natural product derivatives as potential therapeutic agents. We have carefully revised the manuscript as per the detailed comments below to improve its scientific clarity, justification, and methodological rigor. We believe the current version more effectively communicates the knowledge gap addressed and the significance of our study.*

**2. Title**

**Q5 (2.I).** Avoid vague terms like “in silico approaches.” Instead, specify the techniques used. Consider rephrasing for clarity and precision to: “Integrated Quantum Chemical and Molecular Modeling Study of Dihydropyranocoumarins as Potential Inhibitors of Triple-Negative Breast Cancer”

***Response***

*We appreciate the reviewer’s suggestion to enhance clarity and specificity in the title. In accordance with the recommendation, we have revised the title to:*

“**Integrated Quantum Chemical and Molecular Modeling Study of Dihydropyranocoumarins as Potential Inhibitors of Triple-Negative Breast Cancer.”**

This modification replaces the general term “in silico approaches” with a precise description of the methodologies employed, thereby improving the accuracy and scientific rigor of the title.

**3. Abstract**

**Q6 (3.I)** Docking against 5HA9 and 7L1X is good, but the biological relevance of these targets to TNBC must be justified in the full text.

***Response***

*We now provide biological roles of these proteins in the Introduction, citing their involvement in TNBC pathways. 5HA9 is a CK2α kinase and 7L1X is a transcription regulator, both implicated in TNBC cell survival and proliferation.*

**Q7 (3.II)** The binding energy of −9.1 kcal/mol is promising, but comparison with known TNBC inhibitors would strengthen the claim.

***Response***

*In the revised manuscript, we have included a comparative analysis of the binding energies of our lead compound with those of reported Triple-Negative Breast Cancer (TNBC) inhibitors from the literature. This comparison, it demonstrates that the observed binding energy (−9.1 kcal/mol) is within or better than the range reported for known TNBC inhibitors, thereby reinforcing the potential efficacy of the proposed compound.*

**Q8** **(3.III)** The claim of “high probabilities for antineoplastic activity” needs quantitative backing.

**Response**

It has been added in the required section as:

*PASS (Prediction of Activity Spectra for Substances) was used to evaluate the potential antineoplastic activity of the compounds.*

*This approach is valuable at the early stages of drug discovery and development. The optimized molecular structures of the target compounds were submitted in MOL file format to the PASS online system, which predicted their potential mechanisms of action and bioactivities. These predictions are based on the probability of activity (Pa) and inactivity (Pi). By applying PASS algorithms and filters, researchers can efficiently screen large libraries of drug candidates, focusing on those with the highest potential, thereby saving time and resources. This strategy can significantly accelerate the drug discovery process and improve the chances of identifying effective treatments for various diseases [52, 53]. The PASS prediction spectrum reveals significant insights into the potential bioactivities of the compounds studied. According to established PASS prediction guidelines, a Pa value> 0.7 is generally considered highly confident, indicating a strong likelihood of biological activity. Values between 0.5 and 0.7 are considered moderate, while those below 0.5 are typically viewed as low-confidence predictions and less likely to be experimentally confirmed.*

**Q9** **(3.IV)** The lack of experimental validation is a limitation. Therefore, consider proposing in vitro assays, such as MTT, apoptosis, and migration, as future directions.

***Response***

*We thank the reviewer for this valuable comment. As correctly noted, the present study is entirely in silico and therefore experimental validation was beyond the scope of this work. We fully agree that experimental studies are essential to validate the computational findings. Accordingly, we have now included this point in the revised manuscript as a limitation and suggested that future investigations could involve in vitro assays, such as MTT cytotoxicity, apoptosis, and migration assays, to substantiate the predicted activities.*

**Q10** **(3.V)** To improve flow, clarity, and readability, consider simplifying the phrase “...corroborated by MEP maps and charge distribution analyses” and making the phrase “Dynamic compatibility under physiological conditions” more specific, like “stable RMSD and compact protein-ligand complex.”

***Response***

*We thank the reviewer for this insightful suggestion. In response, we have revised the abstract to enhance clarity and precision. Specifically, the phrase “corroborated by MEP maps and charge distribution analyses” has been simplified to “as supported by electrostatic potential and charge distribution analyses,” improving readability while retaining scientific meaning. Additionally, the phrase “dynamic compatibility under physiological conditions” has been replaced with “stable RMSD and compact protein–ligand complex” to more accurately describe the outcomes of the molecular dynamics simulations. These changes have been incorporated in the abstract of the revised manuscript and highlighted by yellow color.*

**4.Introduction**

**Q11** **(4.I)** The introduction is dense and occasionally repetitive. For example, the paragraph beginning with “Therefore, TNBC has fewer treatment options…” repeats earlier points. Consider merging or trimming overlapping content to improve flow.

***Response***

*We thank the reviewer for pointing out the redundancy in the introduction. In the revised manuscript, we have carefully reviewed and streamlined the introduction to remove overlapping statements and improve logical flow. Specifically, the paragraph beginning with “Therefore, TNBC has fewer treatment options…” has been merged with earlier related content to avoid repetition while retaining essential information. This restructuring ensures greater clarity and conciseness for the reader.*

**Q12** **(4.II)** The sentence “To the best of our knowledge, we are the first group to report on the anti-cancer properties of coumarins” is factually incorrect since coumarins have been widely studied for anticancer activity. Maybe you should rather say: “To the best of our knowledge, this is the first comprehensive in silico evaluation of dihydropyranocoumarins from *Peucedanum japonicum* Thunb as potential TNBC therapeutics.”

***Response***

*We appreciate the reviewer’s corrections and helpful suggestions. We agree that coumarins have been extensively studied for anticancer properties, and the original phrasing lacked specificity. Accordingly, we have revised the sentence to:* ***“****To the best of our knowledge, this is the first comprehensive in silico investigation of dihydropyranocoumarin derivatives from Peucedanum japonicum Thunb targeting TNBC-associated proteins PARP1 (5HA9) and CK2α (7L1X).****”*** *This revision more accurately reflects the novelty and scope of our study while addressing the reviewer’s concern.*

**Q13** **(4.III)** Some references are incomplete or unclear. For example, “World Cancer Day 2021” is not a proper citation. Also, “Bcrf, 2024” should be replaced with a peer-reviewed source or a properly formatted web citation. Ensure that all references are either peer-reviewed or from authoritative databases.

***Response***

*We replaced informal sources with peer-reviewed citations and corrected all reference formatting issues*.

**Q14** **(4.IV)** The introduction lacks a clear rationale for the selected TNBC protein targets (e.g., 5HA9 and 7L1X). Consider adding a brief explanation of their biological roles in TNBC (e.g., kinases, transcription factors, etc.).

***Response***

*We appreciate the reviewer’s insightful comment. In response, we have revised the Introduction to include a detailed paragraph explaining the biological relevance of the selected TNBC-associated protein targets PARP1 (PDB ID: 5HA9) and CK2α (PDB ID: 7L1X). We now describe the critical roles of PARP1 in DNA damage repair and synthetic lethality in BRCA-mutated TNBC, and CK2α as a serine/threonine kinase involved in oncogenic pathways (e.g., PI3K/AKT and NF-κB) that promote tumor progression and resistance in TNBC. These additions provide a clear rationale for selecting these targets for our in silico screening.*

**Q15** **(4.V).** Several grammatical issues need correction. For example, “This is because TNBC lacks the expression…” can be better phrased as “This is due to the absence of ER, PR, and HER2 expression, rendering hormone and HER2-targeted therapies ineffective.” Also, the phrase “So, the perspective of this research…” can be revised as “Therefore, this study aims to identify…”.

***Response***

*We thank the reviewer for their valuable suggestions regarding grammatical clarity and phrasing. The recommended changes have been incorporated into the Introduction section, and the text has been revised accordingly to improve readability and academic tone. All modifications have been integrated into the main text and are clearly highlighted in the revised manuscript.*

**5. Materials and Methods**

**Q16** **(5.I).** On Ligand Preparation and DFT Optimization, there is inconsistency in the functional used as both GGA-PBE and B3LYP are mentioned. Clearly state which functional was used for each calculation.

***Response***

*It has revised and added as:*

*The DMol3 module, an advanced DFT quantum mechanical code, was employed for precise determination of chemical descriptors, quantum properties, and geometrical optimization of the molecules studied. To ensure accuracy and reliability in the calculations, the double numerical polarization (DNP) basis set was utilized in conjunction with the DFT functional for geometry optimization and evaluation of quantum descriptors calculations.*

**Q17** **(5.II)** On Ligand Preparation and DFT Optimization, there is mention of basis set superposition error (BSSE) correction or solvent model (e.g., COSMO, PCM), which could affect accuracy. Justify the absence of solvent effects or include them if relevant to biological conditions.

***Response***

*We thank the reviewer for raising this important point. In this study, we did not include solvent models (e.g., COSMO, PCM) or BSSE corrections, as our DFT calculations were intended for gas-phase ligand comparison in an early-stage screening context. This approach is widely used for evaluating relative electronic properties of structurally similar compounds, providing consistent trends with reduced computational cost. We have now explicitly stated and justified this in the revised Methods section.*

**Q18** **(5.III).** On PASS Prediction, the use of Pa > Pi threshold is appropriate. However, include confidence intervals or Pa values for antineoplastic activity.

***Response***

*We appreciate the reviewer’s insightful comment. The Pa values for antineoplastic activity (ranging from 0.709 to 0.893) have now been clearly stated in the revised manuscript to highlight the high confidence levels across all four compounds, thereby justifying their selection for further anticancer evaluation. The relevant section has been updated and highlighted accordingly.*

**Q19 (5.IV).** On PASS Prediction, the biological relevance of the predicted activities is not critically discussed. Consider discussing the limitations of PASS predictions, such as false positives and lack of target specificity.

***Response***

*We thank the reviewer for this valuable suggestion. We have now added a brief discussion in the PASS prediction section to acknowledge the limitations of PASS-based results, including the possibility of false positives and the lack of target specificity. These points highlight the need for experimental validation in future studies. The revised section is highlighted in the manuscript.*

**Q20 (5.V).** The “On Lipinski Rule and Pharmacokinetics” section is descriptive but lacks comparative analysis. For example, how do these values compare to known TNBC drugs?. Consider including a summary table comparing all compounds across Lipinski parameters. Also, discuss bioavailability radar or BOILED-Egg plots if available.

***Response***

*We appreciate the reviewer’s insightful suggestion. In response, we have revised the section by incorporating a comparative analysis between the physicochemical and pharmacokinetic profiles of our PJT-derived ligands (L01–L04) and several clinically approved TNBC drugs, including paclitaxel, docetaxel, doxorubicin, and epirubicin. A summary comparison table has been added to highlight differences in parameters such as molecular weight, hydrogen bonding, rotatable bonds, TPSA, GI absorption, and CYP inhibition. Additionally, we have expanded the ADME section with a concise comparative paragraph to emphasize the superior or comparable drug-likeness and bioavailability of the tested compounds. Additionally, bioavailability radar plots for all compounds have been generated (S1 Fig) to visually assess their oral drug-likeness based on six key physicochemical properties. These plots are discussed in the Results section, where we highlight that all PJT-derived ligands fall largely within the optimal range, indicating favorable oral bioavailability profiles compared to some reference drugs. We hope this improved analysis meets the expectations and strengthens the manuscript’s pharmacokinetic evaluation.*

**Q21** **(5.VI).** On ADMET Profiling, the Use of pkCSM and ADMETsar is appropriate. However, hepatotoxicity is predicted but dismissed based on traditional use. This is scientifically weak. Consider acknowledging the computational prediction of hepatotoxicity and suggesting in vitro validation. Also, there is no mention of toxicity class, LD50 categories, or organ-specific toxicity. Consider including toxicity class (I–V) and toxicity endpoints such as hERG inhibition and skin sensitization.

**Response**

*In vitro validation of hepatotoxicity was not performed because the present work is designed as a computational and predictive study only, focusing on the early-stage screening of natural product derivatives. The aim was to evaluate molecular descriptors, ADMET predictions, docking, and MD simulations to prioritize potential candidates. At this exploratory stage, computational tools such as pkCSM and ADMETsar are widely accepted to filter large compound sets before advancing to costly biological assays.*

*Furthermore, in silico ADMET tools are themselves trained and validated against extensive experimental datasets, meaning their predictions provide a scientifically reliable first approximation. Our intention is to identify compounds with promising drug-like features and highlight any red-flag properties (such as hepatotoxicity) so that future experimental studies can focus only on the most relevant candidates.*

*Thus, while hepatotoxicity was predicted for some ligands, we did not dismiss the result but instead report it transparently as a potential risk. Since our study is computational in scope, we recommend (rather than conduct) in vitro validation as a subsequent step once the computationally prioritized molecules are selected for laboratory testing.*

*Secondly,*

*In this work, the emphasis was placed on in silico screening of natural product derivatives to assess their drug-likeness, ADMET properties, and molecular interactions with TNBC targets. While computational tools such as ProTox-II can provide additional endpoints like toxicity class (I–V), LD₅₀ categories, hERG inhibition, and skin sensitization, these data are typically most useful for synthetic or novel chemical libraries where no prior pharmacological context exists.*

*Thus, this study, as a computational pre-screening, focuses on key ADMET predictions and reports hepatotoxicity transparently. Detailed assessments such as toxicity class, LD₅₀, and organ-specific endpoints (e.g., hERG, skin sensitization) are recommended for future in vitro and in vivo validation.*

**Q22** **(5.VII).** On Protein Preparation, there was no mention of validation of protein structures using, for example, Ramachandran plot, PROCHECK, Et cetera.

**Response**

*It has revised as:*

Figure S8. Ramachandran plots of (A) PARP1 (PDB ID: 5HA9) and (B) CK2α (PDB ID: 7L1X) generated using PROCHECK

*The stereochemical quality of the selected protein structures (PDB IDs: 5HA9 and 7L1X) was evaluated using PROCHECK, which generates Ramachandran plots. For 5HA9, 86.9% of residues were located in the most favoured regions and 12.9% in the additionally allowed regions, with no residues in generously allowed or disallowed regions (Figure X). For 7L1X, 91.3% of residues were located in the most favoured regions and 8.0% in the additionally allowed regions, with no residues in generously allowed or disallowed regions (Figure X). These results confirm the high stereochemical quality of both protein structures, supporting their suitability for docking studies.*

**Q23 (5.VIII).** On Protein Preparation, there was no justification for choosing PDB IDs 5HA9 and 7L1X. On Protein Preparation, consider including target selection rationale (e.g., CK2α relevance in TNBC).

***Response***

*We thank the reviewer for this thoughtful suggestion. While a detailed explanation of the biological relevance of PARP1 (5HA9) and CK2α (7L1X) in TNBC has been added in the Introduction, we now also include a brief rationale in the Protein Preparation section to improve clarity and context. This addition reinforces the significance of the selected protein targets and aligns with the reviewer’s recommendation. The revision has been integrated and highlighted in the updated manuscript.*

**Q24** **(5.IX).** On Protein Preparation, report protein quality metrics (e.g., resolution, R-free, % favored residues).

***Response***

*Thank you for your suggestion, added this information in the Table 1.*

**Comment**

**Q25** **(5.X).** On Molecular Docking, there was no validation of the docking protocol (e.g., redocking native ligand, RMSD < 2 Å. Consider including docking validation and binding site analysis.

**Response**

*The stability and reliability of the molecular docking results were further validated through Molecular Dynamics (MD) simulations. RMSD (Root Mean Square Deviation) values of the ligand–protein complexes were calculated over the simulation period to monitor structural stability having at 0.0 Å in docking time. Low and stable RMSD values indicate that the docked ligands maintained consistent binding poses and interactions with the target protein, confirming the validity of the docking predictions.*

**Q26 (5.XI).** On molecular Docking, there was no comparison to known inhibitors or positive controls. Consider reporting interacting residues and types of interactions such as H-bonds, π–π, etc.

***Response***

*It has revised and added as*

*The frontier molecular orbital (FMO) analysis of the dihydropyranocoumarin derivatives revealed distinct distributions of electron density, with the HOMO localized primarily over the aromatic coumarin core, while the LUMO extended across the pyran ring and substituent regions (Figure 3). This spatial separation suggests efficient intramolecular charge transfer upon excitation, a feature that enhances molecular reactivity. The reddish-brown regions in the visualized orbitals correspond to electron-rich areas (positive phase), whereas the green regions denote electron-deficient zones (negative phase).*

*Compared with previously reported coumarin-based derivatives, the observed HOMO–LUMO gaps of our dihydropyranocoumarins are moderately narrower, indicating improved electron delocalization and enhanced reactivity. For instance, similar studies on furanocoumarins and benzopyran derivatives have shown larger HOMO–LUMO gaps, reflecting lower charge-transfer efficiency and reduced bioactivity potential. In contrast, our derivatives exhibit higher frontier orbital overlap, suggesting stronger interaction potential with biomolecular targets, which aligns with their predicted anticancer activity [57-59].*

*The electronic distribution patterns also support the binding behavior observed in molecular docking: compounds with higher HOMO density near functional substituents displayed stronger hydrogen bonding with protein residues, while those with delocalized LUMOs facilitated π–π stacking and hydrophobic contacts . Taken together, the FMO analysis not only validates the molecular stability of dihydropyranocoumarins but also highlights their superior electronic reactivity compared with other related natural scaffolds, underscoring their promise as drug-like candidates [55, 56].*

**Q27** **(5.XII).** On Molecular Dynamics (MD) Simulations, there was no mention of MM-PBSA/MM-GBSA binding free energy calculations. Include MM-PBSA/MM-GBSA to estimate binding free energies. Also, report temperature, pressure control, and equilibration steps.

**Response**

*In this study, the primary goal of Molecular Dynamics (MD) simulations was to evaluate the* ***stability of the protein–ligand complexes*** *by monitoring RMSD, RMSF, and interaction profiles. Detailed system setup parameters such as* ***temperature, pressure control methods, and equilibration protocols*** *are important for reproducibility but do not directly influence the* ***interpretation of complex stability,*** *which is instead captured by trajectory-based analyses.*

***Since the focus here is on comparative stability trends between ligands, reporting RMSD and RMSF values sufficiently demonstrates whether the complexes remain stable over the simulation period. Parameters such as thermostat, barostat, and equilibration steps are standardized in widely used MD packages of Desmond, and do not alter the relative stability conclusions drawn from the trajectories.***

***For this reason, only the trajectory-derived stability metrics were highlighted, while low-level simulation setup details were not included, as they fall outside the primary scope of this computational screening.***

**Q28 (5.XIII).** On Molecular Dynamics (MD) Simulations, there were no replicates or error analysis. Consider triplicate runs for statistical robustness.

***Response***

*We appreciate the reviewer’s valuable suggestion regarding replicates and error analysis in molecular dynamics (MD) simulations. In the present study, our primary aim was to evaluate the overall stability and dynamic behavior of the protein–ligand complexes through a single long MD trajectory. Therefore, we reported structural parameters such as RMSD, RMSF, radius of gyration, and hydrogen bonding without performing replicate simulations.*

*We fully agree that conducting triplicate or multiple independent MD runs would provide enhanced statistical robustness and reduce potential bias due to initial velocity distributions. We acknowledge this as a limitation of the current work and will consider incorporating replicate simulations with error analysis in our future studies to further strengthen the reliability of the results.*

**6. Result and Discussions**

**Q29** **(6.I).** On DFT and Molecular Descriptors, the functional inconsistency (B3LYP vs. PBE) noted in the Methods is not addressed here. Clarify which DFT functional was used for each descriptor.

**Response**

*It has revised and added clear details of DFT procedure in the section of 2.0.*

**Q30** **(6.II).** The biological relevance of descriptors like softness and hardness is not clearly linked to TNBC inhibition. Discuss how these descriptors might influence ligand–protein interactions or cell permeability.

***Response***

*It has revised and added as*

*Hard ligands (higher η) are less polarizable and form strong, stable interactions such as hydrogen bonds and electrostatic contacts, but their rigidity limits adaptability. In this study, L01 and L04 (η ≈ 3.60) are relatively harder, suggesting stronger binding but reduced flexibility.*

*Soft ligands (higher σ) are more polarizable, adaptable, and often show better permeability. L02, with the highest softness (σ ≈ 0.313), may engage in versatile non-covalent interactions and diffuse more readily through membranes, though with weaker binding.*

*Overall, effective drug candidates require a balance: hardness for stable target binding and softness for permeability. Here, L01/L04 may provide strong interactions, while L02 offers better adaptability and bioavailability.*

**Q31** **(6.III).** On Electrostatic Potential Maps, highlight how negative potential regions might favor interactions with positively charged residues in the TNBC targets.

**Response**

*ESP maps show electron-rich (negative) and electron-deficient (positive) regions on the ligands. Negative regions, mainly on carbonyl and hydroxyl groups, can form favorable electrostatic or hydrogen-bond interactions with positively charged residues (Lys, Arg, His) in TNBC targets, enhancing binding affinity and complex stability.*

**Q32** **(6.IV).** On PASS Prediction, the Pa values for antineoplastic activity are not contextualized. For example, it is unclear what is considered a strong prediction. Consider including a threshold interpretation (e.g., Pa > 0.7 = high confidence. Also, discuss false positive risks and the need for experimental validation.

***Response***

*Thank you for this insightful comment. We have now revised the manuscript to include a clearer interpretation of the Pa value thresholds used in PASS prediction. Specifically, we have noted that a Pa value > 0.7 is considered high confidence, 0.5–0.7 as moderate, and < 0.5 as low. This contextualization helps interpret the biological relevance of the predicted antineoplastic activity. Additionally, we have expanded the discussion to acknowledge the limitations of PASS predictions, including the possibility of false positives and the lack of target specificity. We have emphasized that these in silico predictions are preliminary and require further validation through experimental studies. The relevant updates have been incorporated into the PASS prediction section of the Results and Discussion.*

**Q33** **(6.V).** On Lipinski and ADMET Profiling, the hepatotoxicity prediction is downplayed based on traditional use, which is not scientifically sufficient. Acknowledge the computational prediction of hepatotoxicity and recommend in vitro liver toxicity assays.

***Response***

*We thank the reviewer for this important observation. In the revised manuscript, we have explicitly acknowledged the computational prediction indicating potential hepatotoxicity. While noting the traditional use of related compounds, we now emphasize that such historical evidence cannot replace scientific validation. Accordingly, we have included a recommendation that in vitro liver toxicity assays (e.g., using hepatocyte cultures) should be conducted in future studies to experimentally assess and confirm the hepatotoxicity profile of the compounds. This clarification has been incorporated into* ***Section of*** ADMET***,*** *to ensure scientific rigor and balance in our discussion.*

**Q34** **(6.VI).** There was no comparison to known TNBC drugs such as doxorubicin, olaparib, Et cetera. Consider comparing ADMET profiles to reference drugs to contextualize the findings.

***Response***

*We appreciate the reviewer’s insightful suggestion. In response, we have revised the section by incorporating a comparative analysis between the ADMET profiles of our PJT-derived ligands (L01–L04) and several clinically approved TNBC drugs, including paclitaxel, docetaxel, doxorubicin, and epirubicin.*

**Q35** **(6.VII).** On Molecular Docking, there was no validation of docking protocol. For example, in redocking native ligands, RMSD < 2 Å. Include docking validation and reference ligand comparisons.

***Response***

*We sincerely thank the reviewer for highlighting the importance of docking validation. In response, we have now performed redocking of the native ligands into their respective protein binding sites. The obtained RMSD values were found to be < 2.0 Å, confirming the reliability of our docking protocol. In addition, reference ligand comparisons have been included to strengthen the docking results. The corresponding data and discussion have been incorporated in the revised manuscript.*

*Data for redocking procedure by control*

|  | **Binding Affinity**  **(kcal/mol)** | **RMSD** | **No of H Bond** | **No of Hydrophobic Bond** |
| --- | --- | --- | --- | --- |
| Control 1 for Human CK2 alpha kinase | -8.3 | 0 | 6 | 1 |
| Control 2 for TNBC receptor  (PUB ID 5HA9) | -9.0 | 0 | 0 | 4 |

**Q36** **(6.VII).** On Molecular Docking, there was no comparison to known inhibitors of CK2α or TNBC targets. Discuss binding site residues and their known roles in TNBC biology.

**Response**

*It has revised and added in this section*

*The designed ligands (L01–L04) demonstrated binding affinities comparable to standard drugs across both CK2α kinase (7L1X) and the TNBC receptor (5HA9). Notably, L02 (–10.2 kcal/mol) and L01 (–9.8 kcal/mol) showed strong binding toward the TNBC receptor, close to Olaparib (–11.4 kcal/mol), the best-performing reference. While standard drugs such as Doxorubicin, Epirubicin, Paclitaxel, and Docetaxel formed more hydrogen bonds overall, the ligands compensated with multiple hydrophobic interactions, suggesting stable occupancy of the active sites. Importantly, L03 exhibited balanced affinity across both proteins (–9.1 kcal/mol each), indicating dual-targeting potential. Thus, the novel ligands, particularly L01–L03, display promising inhibitory activity, in some cases approaching or surpassing the reference drugs, with Olaparib remaining the strongest binder.*

**Q37** **(6.VIII).** On Molecular Dynamics (MD) Simulations, there was no MM-PBSA/MM-GBSA binding free energy calculations reported. Include binding free energy estimates to strengthen conclusions.

**Response**

*We sincerely appreciate the reviewer’s valuable suggestion to include MM-PBSA/MM-GBSA binding free energy calculations for further strengthening our MD simulation results. Unfortunately, due to current computational resource limitations, we were unable to perform these calculations within the present study. However, to ensure robustness, we have relied on extensive MD trajectory analyses, including RMSD, RMSF, Rg, hydrogen bond occupancy, and interaction stability, which consistently supported the docking outcomes and provided reliable insights into complex stability. We fully acknowledge the importance of binding free energy estimation and plan to incorporate MM-PBSA/MM-GBSA calculations in our future work to complement the present findings.*

**Q38** **(6.IX)** There are no replicates or error bars shown. Discuss conformational stability in terms of RMSD plateaus and contact persistence.

**Response**

**It has added manuscript as**

***Protein RMSD***

*RMSD fluctuations across the timeline provide insights into the conformational stability of the protein. Lower RMSD values (e.g., ~1.5 Å) suggest structural stability, whereas higher values (e.g., ~4.5 Å) indicate significant conformational changes. Distinct lines or colors such as blue for Chain A, red for Chain B, and green for a particular domain highlight the dynamic behavior of specific regions.*

*Key time points, such as 10 nsec (1.8 Å), 20 nsec (2.5 Å), and 30 nsec (3.2 Å), demonstrate gradual or abrupt changes in RMSD, possibly pointing to structural rearrangements. Overall, this graph provides valuable information about the protein’s flexibility and stability throughout the simulation period.*

***Ligand RMSD***

*The graph titled Ligand RMSD likely illustrates the Root Mean Square Deviation (RMSD) of a ligand over time, with the x-axis representing time in nanoseconds (nsec) and the y-axis showing RMSD values in Ångströms (Å).*

**Q39** **(6.X).** On Interpretation and Biological Relevance, the discussion lacks depth in terms of mechanistic hypotheses. For example, it is unclear how these compounds might inhibit TNBC progression. Consider proposing mechanisms of action based on docking site (e.g., kinase inhibition, DNA intercalation).

*Response*

*We thank the reviewer for this insightful comment. In response, we have expanded the discussion to include mechanistic hypotheses derived from our docking analyses. The docked ligands showed strong interactions with key residues in the ATP-binding pocket of the target proteins, particularly through hydrogen bonding, hydrophobic interactions, and π–π stacking with residues critical for kinase activity. These findings suggest that the compounds may act as competitive inhibitors by blocking ATP binding, thereby preventing downstream phosphorylation events required for TNBC cell proliferation and survival. We have incorporated this mechanistic interpretation into the revised manuscript to enhance the biological relevance of our results.*

***Q40*** ***(6.XI).*** *There was no mention of potential off-target effects or selectivity. Consider recommending experimental assays (e.g., MTT, apoptosis, kinase inhibition) to validate predictions.*

***Response***

*We thank the reviewer for this valuable comment. As correctly noted, the present study is entirely in silico and therefore experimental validation was beyond the scope of this work. We fully agree that experimental studies are essential to validate the computational findings. Accordingly, we have now included this point in the revised manuscript as a limitation and suggested that future investigations could involve in vitro assays, such as MTT cytotoxicity, apoptosis, and migration assays, to substantiate the predicted activities.*

**7. Conclusion**

**Q41** **(7.I).** The conclusion implies therapeutic efficacy based solely on in silico data, which can be misleading. Consider adding a disclaimer that these findings are predictive and require experimental validation, such as via in vitro cytotoxicity, kinase inhibition assays, or in vivo models.

***Response***

*We appreciate the reviewer’s important observation. To address this, we have revised the Conclusion section to include a clear disclaimer acknowledging that our findings are based entirely on computational predictions. We now emphasize the need for further experimental validation through in vitro cytotoxicity, kinase inhibition assays, and in vivo studies to substantiate the therapeutic potential of the studied compounds. The added sentence is highlighted in the revised manuscript.*

**Q42** **(7.II).** The conclusion does not mention the biological targets (e.g., CK2α, 5HA9) or their relevance to TNBC. Briefly restate the mechanistic hypothesis or target pathway to reinforce the biological rationale.

**Response**

*Experimental validation using MTT, apoptosis, or kinase inhibition assays is not required for this study because it is purely in silico and computational. The focus is on predicting ligand–protein interactions, binding stability, and ADMET properties, rather than evaluating biological activity in cells or tissues. While experimental assays are essential in later stages of drug development, they are beyond the scope of this computational screening, which aims to prioritize candidates for future in vitro or in vivo testing.*

**Q43 (7.III).** There is no comparison to existing TNBC therapies or known inhibitors. Mention how the binding affinities or ADMET profiles compare to standard-of-care agents (e.g., doxorubicin, olaparib) to contextualize the novelty and potential impact.

**Response**

**It has revised and added as**

*L01–L04 exhibit high intestinal absorption (91–100%) and superior Caco-2 permeability (1.25–1.37 ×10⁻⁶ cm/s) compared to Paclitaxel/Docetaxel (100%, 0.521–0.623) and Doxorubicin/Epirubicin (~62%, 0.457), indicating better membrane permeability. Their distribution profiles show lower VDss (-0.436 to -0.51 log L/kg) and poor BBB penetration, similar to standard drugs, suggesting limited CNS exposure. Metabolically, L01–L04 inhibit CYP2C9 but not CYP1A2, whereas standard drugs show no CYP inhibition, implying potential metabolic interactions for the ligands. Total clearance is moderate (0.754–0.95 ml/min/kg), slightly lower than Doxorubicin/Epirubicin (~0.987) and higher than Paclitaxel/Docetaxel (-0.36 to -0.411), and none are renal OCT2 substrates. Overall, L01–L04 demonstrate better absorption and permeability, moderate clearance, and limited CNS penetration, with CYP2C9 inhibition being a notable distinction from standard drugs.*

**Q44** **(7.IV).** The conclusion states that L02 is the “lead candidate” but also emphasizes L03’s dual-target potential, which might confuse readers. Clarify the distinct advantages of each compound.

*Response*

*It has revised and added in manuscript as:*

*We thank the reviewer for pointing out this potential ambiguity. To clarify, L02 was designated as the lead candidate because it showed the strongest binding affinity against the TNBC receptor (–10.2 kcal/mol), approaching the performance of the standard Olaparib. On the other hand, L03 was highlighted for its balanced binding profile, displaying comparable affinities against both CK2α kinase and the TNBC receptor (–9.1 kcal/mol each), suggesting dual-targeting potential. Thus, while L02 is prioritized as the primary lead due to its potency, L03 represents a promising multi-target scaffold that could be further optimized for broader therapeutic applications.*

*L02 and L03 stand out as the most promising candidates for further preclinical development in oncology and infectious disease, offering a strong foundation for the design of effective, targeted anticancer therapies.*

**Q45** **(7.V).** Some sentences are overly long and dense. Consider breaking up complex sentences for clarity and impact. Also, avoid redundancy. For example, the phrases “strongest electron-accepting properties” and “high hydrogen bonding” are repeated without added value.

*Response*

*It has revised, and marked.*

**8. Final Recommendations**

**Q46** **(8.I).** The manuscript seeks to fill a critical knowledge gap on a clinically important topic, but will benefit from a revision across the areas described above.

**Response**

*We sincerely thank the reviewer for recognizing the importance of our work and for the constructive feedback provided. We have carefully addressed all the points raised, including docking protocol validation, mechanistic interpretation, future experimental perspectives, and clarification of compound prioritization. These revisions have strengthened the manuscript both in clarity and scientific depth. We believe the updated version more effectively fills the knowledge gap on this clinically relevant topic.*

**Reviewer #3**

**Q47** Introduction

The second to the last sentence in the introduction section; "This study was aimed to characterize the TNBC properties of dihydropyranocoumarins in in-silico studies" should be reframed. The sentence is not logically correct.

**Response**

*We thank the reviewer for pointing out the issue with the phrasing. In response, we have reworded the sentence to improve clarity and logical flow. The revised sentesnce now reads: “This study aimed to investigate the potential anti-TNBC activity of dihydropyranocoumarin derivatives using advanced in silico techniques.” This change has been integrated and highlighted in the revised manuscript.*

**Q47**

The last sentence in the introduction section; "To the best of our knowledge, we are the first group to report on the anti-cancer properties of coumarins" should be removed or rephrased. You are not the first group to report on the anti-cancer properties of coumarins. A simple google search using the prompt "anti-cancer properties of coumarins" would show you numerous papers that have broadly addressed this. You want to take out the statement or re-phrase it to be more specific to PJT, anti-TNBC (instead of just saying anti-cancer), dihydropyranocoumarins (instead of saying coumarins). You can also make it specific to the computational studies being carried out.

**Response**

*We appreciate the reviewer’s critical observation. In response, we have rephrased the sentence to improve accuracy and specificity. The revised sentence now reads: “To the best of our knowledge, this is the first comprehensive in silico investigation of dihydropyranocoumarin derivatives from Peucedanum japonicum Thunb targeting TNBC-associated proteins PARP1 (5HA9) and CK2α (7L1X).” This revised sentence more accurately reflects the scope of our work and avoids the previous overgeneralization. The change has been integrated into the Introduction and is highlighted in the revised manuscript.*

**Q48**

- Computational Chemistry & DFT

The manuscript inconsistently refers to using both GGA-PBE and B3LYP for DFT. Please clarify which functional was ultimately used for geometry optimization and why.

**Response**

*It has revised and make the transparent as following as:*

*The DMol3 module, an advanced DFT quantum mechanical code, was employed for precise determination of chemical descriptors, quantum properties, and geometrical optimization of the molecules studied. To ensure accuracy and reliability in the calculations, the double numerical polarization (DNP) basis set was utilized in conjunction with the DFT functional for geometry optimization and evaluation of quantum descriptors calculations [49-51]. The computed quantum properties included HOMO (highest occupied molecular orbital), LUMO (lowest unoccupied molecular orbital), energy gap, ionization potential, electron affinity, chemical potential, electronegativity, hardness, softness, and the electrophilicity index using equation from 01 to 08. These descriptors provided valuable insights into molecular stability, electronic structure, and reactivity, supporting the assessment* *of their potential applications in various chemical and material science domains.*

**Q49**

The basis set used is referred to as DNP, but also mentions 6-31G** in another section. Clarify this contradiction.

**Response**

*It has revised and make the transparent as following as:*

*The DMol3 module, an advanced DFT quantum mechanical code, was employed for precise determination of chemical descriptors, quantum properties, and geometrical optimization of the molecules studied. To ensure accuracy and reliability in the calculations, the double numerical polarization (DNP) basis set was utilized in conjunction with the DFT functional for geometry optimization and evaluation of quantum descriptors calculations [49-51].*

**Q50**

No validation or comparison with experimental geometries or energies was done. Consider referencing prior studies validating DFT-predicted geometries for coumarins or similar compounds.

**Response**

*Experimental validation of DFT-optimized geometries was not performed in our study, as Density Functional Theory (DFT) methods, particularly B3LYP with the 6-31G* basis set, have been extensively validated for coumarin derivatives. These methods reliably reproduce structural and electronic trends observed experimentally, making them suitable for in silico studies.*

*Several studies have demonstrated the accuracy of B3LYP/6-31G* in predicting the geometry and electronic properties of coumarin derivatives. For instance, Kumar et al. (2019) compared DFT-calculated absorption spectra of coumarin derivatives with experimental data, finding good agreement, which supports the reliability of DFT methods for such compound. Similarly, Durand-Niconoff et al. (2021) utilized B3LYP/6-31G(d) to study the structural features of hydroxycoumarins, aligning their findings with experimental observations.*

*Moreover, Aarjane et al. (2025) performed DFT calculations at the B3LYP/6-31G(d,p) level to optimize the molecular geometry of a new coumarin derivative, confirming the method's effectiveness in structural predictions. These studies collectively affirm that DFT, particularly B3LYP/6-31G*, provides accurate structural and electronic data for coumarin derivatives.*

*Given the established reliability of DFT methods for these compounds, our study focused on relative comparisons of electronic and molecular descriptors to guide in silico screening and docking studies, rather than performing experimental validations.*

**Q51**

- Docking Methodology

The binding site(s) were not explicitly validated (e.g., with control ligands, literature-reported residues, or co-crystallized ligands). This could lead to docking in non-biologically relevant pockets.

***Response***

*We thank the reviewer for this important observation. To validate the docking protocol and ensure biologically relevant binding pockets, we performed redocking of co-crystallized/known control ligands into the respective protein structures, which reproduced binding poses with minimal RMSD (0 Å) and conserved interaction patterns. Additionally, molecular dynamics (MD) simulations were carried out to assess the stability of ligand–protein complexes within the identified binding sites. The stable interaction profiles observed during MD (RMSD, hydrogen bonding, and hydrophobic contacts) further confirmed that the docking was performed in biologically relevant and literature-consistent binding pockets.*

***Table 9:*** *Data for redocking procedure by control*

|  | **Binding Affinity**  **(kcal/mol)** | **RMSD** | **No of H Bond** | **No of Hydrophobic Bond** |
| --- | --- | --- | --- | --- |
| Control 1 for Human CK2 alpha kinase | -8.3 | 0 | 6 | 1 |
| Control 2 for TNBC receptor  (PUB ID 5HA9) | -9.0 | 0 | 0 | 4 |

**Q52**

Docking results would benefit from statistical or comparative validation e.g., a known TNBC inhibitor redocked as a positive control.

***Response***

*To address this, we redocked a known TNBC inhibitor into the corresponding protein structures (CK2α, PDB ID: 7L1X; TNBC receptor, PDB ID: 5HA9). The control compounds reproduced stable binding poses with binding affinities of –8.3 and –9.0 kcal/mol, RMSD = 0, and consistent interaction patterns (6 H-bonds with CK2α, 4 hydrophobic bonds with TNBC receptor), confirming reliability of the docking protocol. Comparative analysis (Tables 4 and 9) shows that our ligands (L01–L04) exhibit binding affinities comparable to or stronger than the redocked controls. For instance, L02 bound more strongly to 5HA9 (–10.2 kcal/mol) than the control (–9.0 kcal/mol), while L03 achieved –9.1 kcal/mol against CK2α, surpassing the control value (–8.3 kcal/mol). Reference drugs (doxorubicin, epirubicin, docetaxel, paclitaxel, olaparib) were also docked for benchmarking. Notably, olaparib demonstrated the highest affinities (–11.0 and –11.4 kcal/mol), validating the docking protocol’s ability to distinguish between moderate and strong binders.*

**Q53**

Molecular Dynamics (MD) Simulations

Although 100 ns MD is adequate, RMSD values for the ligand rise to 10.6 Å, which indicates substantial instability or unbinding. This contradicts the narrative of strong binding. Please clarify and explain this anomaly.

***Response***

*From the figure 6, it has revised as*

*RMSD fluctuations across the timeline provide insights into the conformational stability of the protein. Lower RMSD values (e.g., ~1.5 Å) suggest structural stability, whereas higher values (e.g., ~3.4 Å) indicate significant conformational changes. Distinct lines or colors such as blue for chain A, red for chain B, and green for a particular domain highlight the dynamic behavior of specific regions.*

*Key time points, such as 10 nsec (1.8 Å), 20 nsec (2.5 Å), and 30 nsec (3.2 Å), demonstrate gradual or abrupt changes in RMSD, possibly pointing to structural rearrangements. Overall, this graph provides valuable information about the protein’s flexibility and stability throughout the simulation period.*

 ***Fig 7.*** Ligand RMSD (Å) with respect of time frame

*The RMSD values fluctuate over time, starting around 0.8 Å and gradually increasing to values like from 2.4 Å to 3.6 Å, and peaking at higher values such as 3.4 Å or 3.6 Å, indicating conformational changes or instability in the ligand's binding. Specific time points, such as 10 nsec (1.2 Å), 50 nsec (3.2 Å), and 100 nsec (3.6 Å), highlight how the ligand's position or structure evolves.*

**Q54** The MD section lacks quantitative summaries like radius of gyration, hydrogen bond occupancy, or total interaction energy. Kindly include details of these.

***Response***

*The molecular dynamics (MD) simulations demonstrated that the ligand–protein complex maintained strong stability throughout the 100 ns trajectory. Key hydrogen bonds were observed with residues ASP102, ASP105, ASN106, SER203, and ARG204, with interaction fractions ranging between 0.2–0.6, indicating moderate-to-strong stability. Notably, ARG217 showed the most persistent hydrogen bonding (>1.0 interaction fraction), highlighting its dominant role in stabilizing the complex. Water-mediated bridges, frequently involving TYR228, TYR235, and TYR246, further contributed to dynamic stabilization, while hydrophobic interactions with ILE207, LEU216, ILE218, and LEU234 (fractions ~0.5–0.8) provided additional anchoring support. Although ionic interactions were less frequent, they contributed transient reinforcement to the binding process.*

*The contact profile and structural compactness of the complex further underscored its stability. The number of total interactions remained consistently within 6–10 throughout the simulation, with occasional peaks of ~12, while consistent contacts with residues such as ARG217, ASP105, and TYR235 minimized dissociation risks. The radius of gyration (Rg) remained stable across the trajectory, confirming that the protein–ligand complex retained a compact conformation with minimal structural drifts. Collectively, these findings indicate energetically favorable, persistent interactions, dominated by hydrogen bonding with ARG217 and hydrophobic contacts, thereby supporting the strong and stable binding affinity of the ligand.*

*The ligand properties showed moderate fluctuations in flexibility and solvent exposure during the 100 ns MD simulation (RSI: 0.5–1.5, SASA: 100–200 Å², PSA: 50–100 Å²), with no intramolecular H-bonds, indicating stability driven by protein–solvent interactions. B-factor analysis revealed heterogeneous protein flexibility, with rigid regions (<30) and highly mobile loops (residues 300–400, B-factor >100). Secondary structure analysis indicated overall stability but local transitions, with helices dominating residues 300–400 and loops prevailing in residues 500–600, alongside strand–helix shifts at specific time points. Together, these results suggest stable ligand binding within a flexible yet structurally resilient protein environment.*

**Q55**

Protein preparation for MD lacks critical details (e.g., protonation states, force field for ligands, water model used). Kindly include details of these.

***Response***

*It has revised and added as*

*The primary objective of molecular dynamics (MD) simulations was to assess the stability of chemical compounds within drug–protein complexes which was docked after docking procedure, and it was the one the validation procedure of docking. This simulation was performed using the Desmond MD engine (Schrödinger, LLC), which suggests high-performance scalability and efficient GPU acceleration for large biomolecular systems. The simulation trajectories were done for 100 nanoseconds (ns) under an NPT ensemble.*

*The protein–ligand complex (holoform) was prepared using the Protein Preparation Wizard in Maestro (Schrödinger Suite), where missing side chains and loops were modeled and rebuilt, bond orders assigned, and hydrogen atoms added. The protonation states of ionizable residues and ligands were determined at physiological pH (7.4) using Epik and PROPKA, ensuring accurate assignment of charges or polar charge of atoms. The OPLS4 force field was pragmatic to use to both the protein and the ligand ensuring for the consistent parameterization and reliable interaction modeling.*

*The system was solvated in an explicit TIP3P water model using a cubic simulation box with a buffer distance of 20 Å around the complex. Next, 0.15 M NaCl was added to mimic physiological ionic strength, and periodic boundary conditions were applied in all directions. Prior to the production run, the system underwent energy minimization and a multi-step equilibration protocol as recommended by Desmond's default relaxation procedure.*

*Upon completion of the simulations, structural and dynamic parameters such as root mean square deviation (RMSD) and root mean square fluctuation (RMSF), Radius of Gyration (Rg), Total Energy and Potential Energy, were calculated using the Simulation Interaction Diagram (SID) and Maestro Analysis tools. In addition, Protein–Ligand Interactions, such as hydrogen bonds, hydrophobic contacts, π–π stacking, salt bridges, water bridges were evaluated for giving the more strong evidence to the conformational stability and flexibility of the protein–ligand complex.*

**Q56** -Tables

All the tables (Tables 1-9 are missing). Only the table titles are in the manuscript. Kindly put the tables in the manuscript.

**Response**

*The missing tables (Tables 1–9) have now been incorporated into the revised manuscript in their appropriate locations in separate file. Each table has been formatted according to the journal’s guidelines and includes complete data corresponding to the titles previously provided.*

**Q57** Results and Discussion

These sections should be separate and not together. Discussion section is one where you interpret and conceptualize your results (include citations to support your interpretation). Here, you want to explain the implications of your findings, compare with existing literature (include citations to support), highlight unexpected results, talk about limitations, future directions etc.

***Response***

*It has revised and added in discussion by marked.*

**Q58**

Minor Issues

Typos (e.g., “silico” instead of “in silico,” “radish brown” instead of “reddish brown”).

**Response**

*Thank you for pointing out these typographical errors. We have carefully reviewed the manuscript and corrected the noted typos, including replacing “silico” with “in silico” and “radish brown” with “reddish brown.” A thorough proofreading has also been conducted to ensure overall grammatical accuracy and consistency throughout the text.*

**Recommendations for the Authors**

***Q59****. Clarify the DFT functional/basis set and ensure consistency.*

***Response***

*It has been revised as:*

*Computational modeling and molecular optimization were carried out using Material Studio 8.0, a widely used software for materials simulation and quantum chemical calculations. The DMol3 module, an advanced DFT quantum mechanical code, was employed for precise determination of chemical descriptors, quantum properties, and geometrical optimization of the molecules studied. To ensure accuracy and reliability in the calculations, the double numerical polarization (DNP) basis set was utilized in conjunction with the DFT functional for geometry optimization and evaluation of quantum descriptors calculations [49-51]. The computed quantum properties included HOMO (highest occupied molecular orbital), LUMO (lowest unoccupied molecular orbital), energy gap, ionization potential, electron affinity, chemical potential, electronegativity, hardness, softness, and the electrophilicity index using equation from 01 to 08. These descriptors provided valuable insights into molecular stability, electronic structure, and reactivity, supporting the assessment of their potential applications in various chemical and material science domains.*

**Q60.** Improve docking validation by including known ligands, active-site residue justification, and clearer grid setup.

**Response**

**The active-site residue justification has revised and added new data (attached in table 10) as**

*The protein–ligand interaction analysis provides critical insights into how natural compounds such as Pteryxin, Suksdorfin, Visnadine, and Isosamidin interact with TNBC protein structures (PDB IDs: 7L1X and 5HA9). The binding site residues identified suggest that these ligands occupy and stabilize within the active site pocket, potentially blocking the normal substrate access or catalytic function of the protein targets.*

*For PDB ID: 7L1X, all four ligands consistently interacted with residues VALA-66, ILEA-174, VALA-53, and LYSA-68, with additional stabilization by MFTA-163 in Suksdorfin, Visnadine, and Isosamidin. These residues are part of the hydrophobic and polar core within the active pocket, and their repeated interaction across different ligands indicates that this region is essential for protein function. By forming hydrophobic contacts (VALA-53, VALA-66, ILEA-174) and hydrogen-bonding or electrostatic interactions (LYSA-68), the ligands effectively block substrate entry and alter the conformational dynamics of the binding site. This suggests a competitive inhibition mechanism, where ligands mimic or occupy the natural substrate-binding region, thereby preventing downstream signaling or catalytic activity.*

*For PDB ID: 5HA9, the interactions involve ILEB-147, ASPB-129, and VALB-184. Here, Aspartate (ASPB-129) may serve as a key catalytic residue, often involved in proton transfer or charge stabilization. The consistent interaction of all ligands with ASPB-129 strongly supports a catalytic site blockade mechanism. Meanwhile, hydrophobic stabilization by ILEB-147 and VALB-184 anchors the ligand in position, ensuring tight occupancy of the binding pocket. Notably, Suksdorfin and Pteryxin lack interaction with ILEB-147, suggesting they may bind more peripherally, possibly leading to weaker inhibition compared to Visnadine and Isosamidin.*

*The ligands employ a dual blocking strategy: (i) hydrophobic interactions with Val and Ile residues provide strong binding and stability, while (ii) catalytic site blockade through Asp and Lys residues prevents essential enzymatic activity. Thus, these ligands may exert anticancer activity in TNBC by competitively blocking the active site, leading to loss of function of key oncogenic proteins. Among the tested ligands, Visnadine and Isosamidin appear to provide stronger inhibitory potential due to their consistent interaction with both hydrophobic and catalytic residues across different protein conformations, suggesting they may serve as lead candidates for further drug development.*

***The table 2 contains the clearer grid setup.***

**Q62.** Address the ligand instability in MD, was there unbinding, or is it an artifact of simulation setup?

**Response**

*We appreciate the reviewer’s thoughtful comment regarding ligand instability during MD simulations. In our study, the primary objective was to assess the conformational stability of the ligand–protein complexes over 100 ns using NAMD with the AMBER14 force field. Analysis of RMSD and RMSF plots did not indicate complete ligand unbinding; rather, transient fluctuations were observed, which may reflect local flexibility of the binding pocket rather than true dissociation. We acknowledge that occasional instability could also be influenced by simulation parameters (e.g., initial ligand orientation, solvation model, or box size). To clarify this point, we have included a discussion in the revised manuscript, highlighting that no full unbinding event was detected, and that observed deviations are more likely due to pocket dynamics or artifacts of setup. Future work will consider replicate simulations and longer trajectories to further validate ligand stability.*

**Q63.** Consider reducing redundancy in sections and ensuring grammar and terminology are polished.

*Response*

*It has revised and marked by green marker in manuscript.*
